# Disproportionality analysis of methylphenidate-associated adverse events in pediatric patients using the FDA Adverse Event Reporting System: overall signals and stratification by gender and dosage form

**DOI:** 10.3389/fpsyt.2026.1896696

**Published:** 2026-08-12

**Authors:** Zhonglan Wang, Yan Gao, Jing Zhang, Yu Wang, Zhe Wang, Yichi Zhang, Lina Hao

**Affiliations:** Department of Pharmacy, Children’s Hospital Affiliated to Shandong University, Jinan, China

**Keywords:** methylphenidate, pediatric patients, adverse drug events, FAERS, gender differences, dosage form

## Abstract

**Background:**

Methylphenidate is widely prescribed for the clinical management of pediatric patients with attention-deficit/hyperactivity disorder (ADHD), yet its safety profile remains a subject of ongoing concern. This study aims to characterize the real-world safety profile of methylphenidate in children and adolescents using the FDA Adverse Event Reporting System (FAERS) database.

**Methods:**

Adverse drug event (ADE) reports for individuals aged 6–17 years were extracted from the FAERS database via OpenVigil 2.1. Disproportionality analyses were performed to detect methylphenidate-associated safety signals, including overall signal detection as well as subgroup analyses stratified by gender and dosage form.

**Results:**

From 8,585 reports, methylphenidate-associated adverse drug reaction (ADR) signals involved 16 System Organ Classes (SOCs), most frequently psychiatric disorders; general disorders and administration site conditions; and nervous system disorders. Several potential signals not listed in current drug labels were identified, including abnormal behavior, disturbance in social behavior, and coronary artery dissection. Gender-specific analyses revealed 86 positive signals in males and 90 in females, with 41 male-exclusive and 45 female-exclusive signals. Male-exclusive signals included arrhythmia, coronary artery dissection, and acute myocardial infarction. Female-exclusive signals included tachycardia and application site reactions. Among positive signals detected in both genders, female patients showed higher reporting of application site erythema, agitation, application site pruritus, precocious puberty, and application site discolouration, whereas male patients showed higher reporting of aggression, tics, and growth retardation. Dosage-form-specific analyses showed that the immediate-release formulation was associated with acute, severe signals (e.g., sudden death, syncope). The oral extended-release formulation accounted for the largest number of unique signals (n = 63) in this dataset, although this should be interpreted with caution given differences in reporting volume across formulations. The transdermal patch was predominantly associated with application site reactions (e.g., erythema, pruritus).

**Conclusion:**

This study identified overall, gender-, and dosage-form-specific ADR signals for methylphenidate in pediatric patients, including signals not currently listed in drug labels. Distinct ADR signal profiles were observed across gender and dosage-form strata. Individualized pharmacovigilance strategies may be informed by these subgroup-specific ADR signal profiles. These findings contribute to the current understanding of methylphenidate-associated ADE in children and adolescents and warrant further validation in future studies.

## 1 Introduction

Due to the unique physiological characteristics of pediatric populations and the immature development of their organs and functional systems, children may be at a higher risk of drug toxicity and more susceptible to adverse drug reactions (ADRs) compared to adults (1, 2). Several studies (3, 4) have shown that methylphenidate accounts for a higher proportion of adverse drug events (ADEs) reported in children and adolescents through the Spontaneous Reporting System (SRS). Methylphenidate is a central nervous system stimulant and a first-line pharmacotherapy for Attention-Deficit/Hyperactivity Disorder (ADHD) (5, 6). It is also indicated for the management of narcolepsy (7, 8). Common ADRs associated with methylphenidate include tachycardia, insomnia, anxiety, weight loss, decreased appetite, dry mouth, nausea, and abdominal pain (9). Public and professional concerns regarding the safety of methylphenidate primarily focus on severe ADR, including serious cardiovascular events, growth suppression, and psychiatric disorders (10). Methylphenidate is indicated for the treatment of ADHD in children and typically requires a treatment duration ranging from one to several years (11). In recent decades, the global utilization of methylphenidate has risen markedly (12, 13). This trend has been accompanied by an increase in its abuse and misuse, as well as an elevated risk of ADR (14). Due to the uncertainty of benefit-risk ratio of long-term methylphenidate use, it has not been included in the WHO Model List of Essential Medicines (15). Therefore, it is of particular importance to deeply explore the ADRs of methylphenidate in pediatric patients in the real world, especially the rare and serious ones, in order to evaluate its long-term safety.

The safety profile of methylphenidate in children, as obtained from clinical trials, remains limited due to ethical considerations and challenges in patient recruitment. Utilizing SRS to identify ADR signals of marketed drugs in real-world pediatric populations represents a critical approach for supplementing existing safety information. The FDA Adverse Event Reporting System (FAERS) (16) is a critical pharmacovigilance tool for post-marketing safety surveillance. It aggregates ADE reports not only from the United States, but also from other countries worldwide. With a substantial volume of data, the FAERS database provides detailed information on patients, drugs, adverse events, and other relevant variables, and is freely accessible to users.

Notably, gender differences in methylphenidate pharmacokinetics (17) and efficacy (18) have been reported, yet few studies have systematically investigated gender-specific ADR profiles in pediatric populations. Similarly, dosage-form-specific safety profiles remain inadequately characterized. This study therefore conducted a disproportionality analysis using the FAERS database to identify real-world ADR signals associated with methylphenidate in pediatric patients, with particular attention to gender- and dosage-form-specific differences. The findings may inform safety warnings and support individualized pharmacovigilance strategies for methylphenidate use in children and adolescents.

## 2 Methods

### 2.1 Data sources

ADE reports were extracted from the FAERS database using the OpenVigil 2.1 platform, a pharmacovigilance tool that facilitates data cleaning, structuring and analysis (19). OpenVigil 2.1 standardizes drug names (generic names, brand names, and abbreviations) to unique identifiers via DrugBank and Drugs@FDA (20). Using the OpenVigil 2.1 web-based interface (https://openvigil.sourceforge.net/), we retrieved reports from January 1, 2004 to March 31, 2025, limiting the search to the generic name “methylphenidate” and the age range of 6–17 years. Only cases with the most recent individual safety report (ISR) and reports in which methylphenidate was identified as the primary suspected (PS) drug were included in this analysis. The data extraction and filtering process is detailed in **Supplementary Table 1**. The retrieved reports contained detailed information, including ISR number, outcomes, drug names, role codes, routes, dosages, indications, events, case IDs, patient gender, reporter country, and age in report.

### 2.2 Data processing and statistical analysis

To enhance the reliability and robustness of signal detection, we performed a multi-method disproportionality analysis using four complementary statistical algorithms: the Reporting Odds Ratio (ROR), the Proportional Reporting Ratio (PRR), the Bayesian Confidence Propagation Neural Network (BCPNN), and the Multi-item Gamma Poisson Shrinker (MGPS) (21). ROR and PRR are frequentist metrics offering high sensitivity but limited specificity, whereas BCPNN and MGPS employ Bayesian shrinkage to stabilize estimates for rare events, enhancing specificity and reducing false positives (22, 23).

Adverse event terms were mapped to Preferred Terms (PTs) using the Medical Dictionary for Regulatory Activities (MedDRA), Version 27.0. The classification of ADE was performed using the System Organ Class (SOC). Positive ADR signals were defined as those meeting all of the following criteria (24): (1) number of reports ≥ 3; (2) the lower bound of the 95% confidence interval (CI) of ROR > 1; (3) PRR ≥ 2 and the chi-square value (χ^2^) ≥ 4; (4) the lower bound of 95% CI of the information component (IC025) > 0; (5) the lower bound of 95% CI of empirical Bayesian geometric mean (EBGM05) > 2. Detailed equations and criteria for each algorithm are provided in **Supplementary Tables 2**, **S3**. Higher values of ROR, PRR, IC025, and EBGM05 indicate stronger ADR signals, reflecting a greater statistical association between methylphenidate and the ADE. Based on the signal detection criteria of the ROR, PRR, BCPNN and MGPS methods, PTs with positive signals were identified and selected. An ADR is defined as a harmful or unpleasant reaction resulting from the use of a medicinal product (25). Therefore, we excluded signals that did not represent clinical adverse reactions or were unrelated to drug exposure. The flow of data processing and statistical analysis is detailed in **Supplementary Table 1**.

To investigate gender-related differences in methylphenidate-related ADR signals among pediatric patients, four algorithms (ROR, PRR, BCPNN, and MGPS) were employed to identify positive ADR signals in male and female pediatric patients. Subsequently, for positive signals detected in both genders, further gender-specific analyses were conducted using 2 × 2 contingency tables with a modified ROR method (**Supplementary Table 4**). An ROR > 1 with the lower bound of its 95% CI > 1 and a chi-square *P* value < 0.05 indicates a higher reporting frequency in boys. Conversely, an ROR < 1 with the upper bound of its 95% CI < 1 and a *P* value < 0.05 indicates a higher reporting frequency in girls (26).

To investigate dosage-form-specific ADR patterns associated with methylphenidate, ADE reports from the FAERS database were classified by Pharmaproduct (brand name) into three mutually exclusive categories: oral immediate-release, oral extended-release, and transdermal extended-release formulations, based on the classification described in the NCBI StatPearls resource (27). Reports that did not match any of the listed brand names, or for which Pharmaproduct information was missing or could not be reliably interpreted, were classified as “Uncertain” and included in the overall and gender-stratified analyses but excluded from dosage-form-specific subgroup analyses. The list of Pharmaproducts identified in the 8,585 reports and assigned to each formulation group is provided in **Supplementary Table 1**. The ROR, PRR, BCPNN, and MGPS algorithms were applied to detect positive ADR signals for each formulation, followed by comparative analysis of signal distributions at the PT and SOC levels.

To assess the robustness of the identified signals and to control for false-positive findings arising from multiple comparisons, we performed a sensitivity analysis using Bonferroni correction (28). The total number of comparisons (N) was defined as the sum of positive signals identified across all analysis strata, including overall, gender-stratified, and dosage-form-stratified analyses. The corrected significance threshold was set at α = 0.05/N. Bonferroni-adjusted *P*-values were calculated as *P*_adj = *P*_raw × N (capped at 1.0). Signals with adjusted *P*-values < 0.05 were considered robust to multiple comparisons.

All statistical analyses and data visualization were performed using R software (version 4.5.0) and Microsoft Excel.

This study was reported in accordance with the READUS-PV guidelines, with relevant principles from RECORD-PE considered where applicable, to ensure transparent reporting of disproportionality analyses (29, 30).

## 3 Results

### 3.1 Descriptive analysis

A total of 8,585 ADE reports with methylphenidate as the PS drug were retrieved from the FAERS database. The basic characteristics of these reports are summarized in **Table 1**. Regarding gender distribution, males accounted for 72.34% of all reports, while females accounted for 25.45%. Regarding dosage form distribution, oral immediate-release formulations accounted for 5.70%, oral extended-release formulations for 24.59%, transdermal extended-release formulations for 24.24%, and the dosage form was undetermined for 45.47% of reports.

**Table 1:** Characteristics of ADE reports for methylphenidate (Q1–2004 to Q1 2025).

Characteristics	Case number	Case proportion(%)
Gender		
Male	6210	72.34
Female	2185	25.45
Unknown	190	2.21
Reported countries(top 5)		
United States	5415	63.08
United Kingdom	400	4.66
Germany	283	3.30
Turkey	257	2.99
Japan	228	2.66
Dosage Forms		
Oral Immediate-Release	489	5.70%
Oral Extended-Release	2111	24.59%
Transdermal Extended-Release	2081	24.24%
Uncertain	3904	45.47%
Serious outcome		
Hospitalization (initial or prolonged)	1105	12.87
Life-threatening	192	2.24
Disability	179	2.09
Death	146	1.70

**Figure 1:**
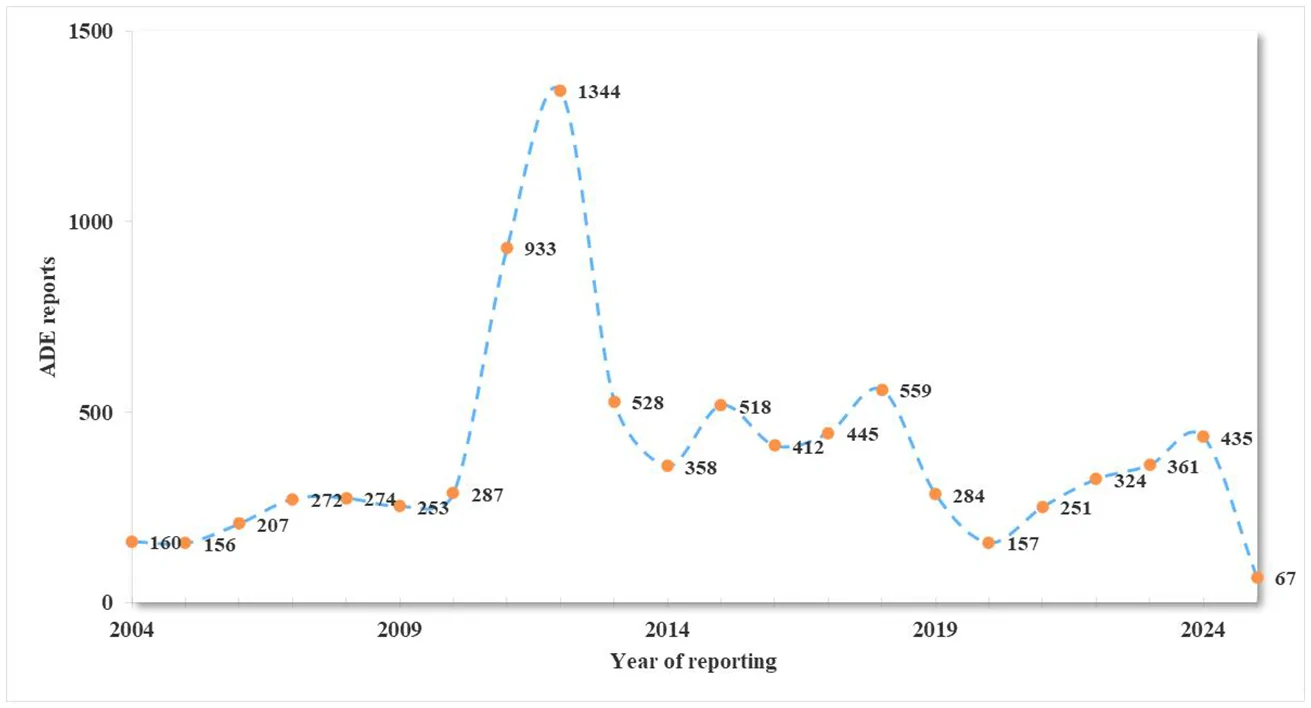
Number of ADE reports per year for methylphenidate as PS.

As shown in the annual trend of ADE reporting for methylphenidate, there was a gradual increase from 2004 to 2010, followed by a sharp rise in 2011 and 2012. After that, the volume of reports declined to levels comparable to those observed prior to the increase, as illustrated in **Figure 1**.

**Figure 2:**
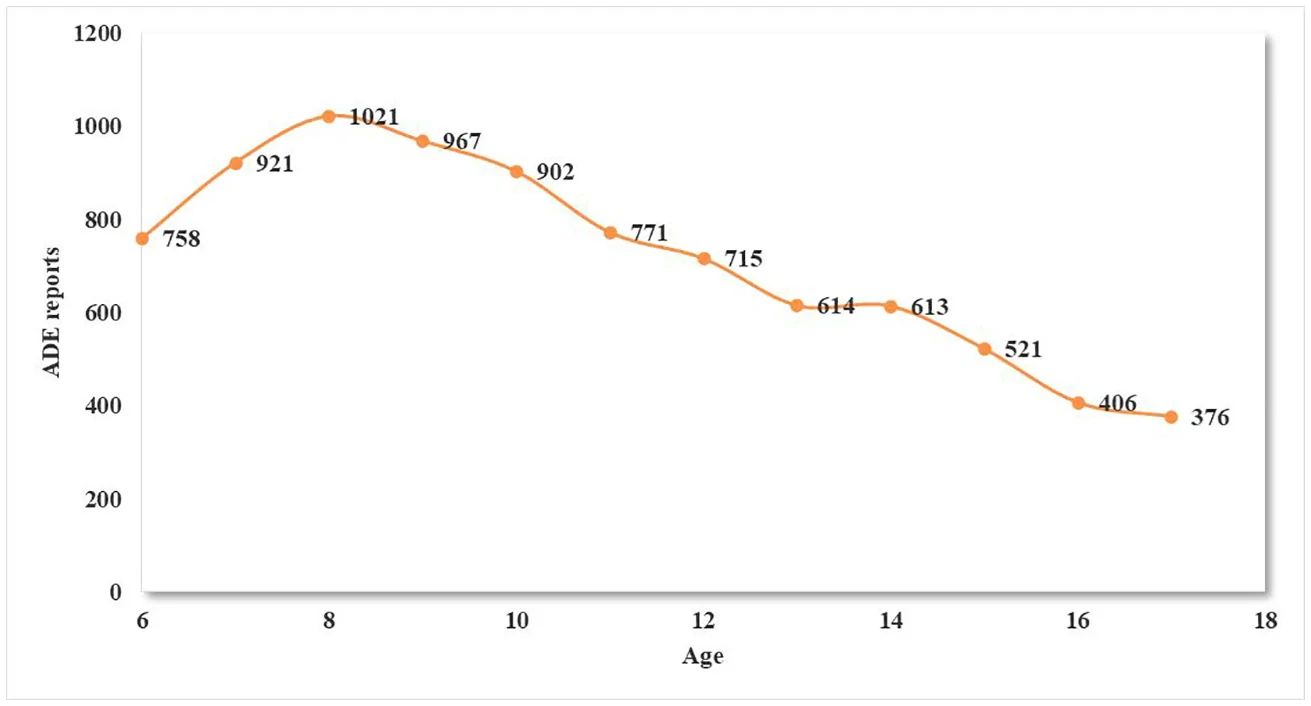
Number of ADE reports for methylphenidate among individuals aged 6 to 17 years.

According to the age group distribution in the ADE reports, the 8-year-old group had the highest number of reported cases (n = 1021), followed by the 9-year-old (n = 967) and 7-year-old (n = 921) groups. ADE reports associated with methylphenidate were predominantly concentrated among children aged 6 to 11 years, accounting for 62.20% of the total reports, as shown in **Figure 2**.

### 3.2 Overall PTs analysis and SOCs distribution

The ROR, PRR, BCPNN, and MGPS algorithms were applied to detect positive ADR signals of methylphenidate in the overall pediatric patient cohort. After excluding the PTs associated with product issues, medication errors, and drug indications, a total of 132 positive signals were identified as shown in **Supplementary Tables 1, S5**. In terms of reporting frequency, the top five ADR signals for methylphenidate in the pediatric population were application site erythema (n = 418), abnormal behavior (n = 403), aggression (n = 389), decreased appetite (n = 372), and insomnia (n = 307). The top 20 most frequently reported ADR signals are presented in **Table 2**. When ranked by signal intensity based on the ROR values, the top five ADR signals were coronary artery dissection (n = 24, ROR = 767.07), dermal absorption impaired (n = 6, ROR = 191.37), Huntington’s disease (n = 4, ROR = 127.55), application site erosion (n = 17, ROR = 108.58), and application site erythema (n = 418, ROR = 106.85). The top 20 ADR signals ranked by ROR values are shown in **Table 3**. Notably, ADR signals that have not been included in the current drug labels are marked with an asterisk (*) in **Tables 2**, **3**. To further characterize the coronary artery dissection signal (n = 24, ROR = 767.07), we extracted the available case-level details from the FAERS database (**Supplementary Table 6**). Among these 24 cases, 23 had no concomitant medications, and the remaining case reported five additional drugs (eptifibatide, aspirin, bisoprolol, lisinopril, and prasugrel) with the role code “Interacting”. Furthermore, 15 of the 24 cases were also reported with acute myocardial infarction and 9 with myocardial infarction.

**Table 2:** Top 20 PTs of methylphenidate based on frequency.

PT	SOC	Case reports	ROR(95%CI)	PRR(χ^2^)	IC025	EBGM05
Application site erythema	General disorders and administration site conditions	418	106.85(87.70, 130.18)	101.70 (9964.49)	4.37	21.22
Abnormal behavior*	Psychiatric disorders	403	3.94(3.55, 4.38)	3.80 (753.73)	1.65	3.21
Aggression	Psychiatric disorders	389	3.34(3.00, 3.72)	3.23 (553.48)	1.44	2.77
Decreased appetite	Metabolism and nutrition disorders	372	4.07(3.65, 4.54)	3.94 (734.07)	1.68	3.30
Insomnia	Psychiatric disorders	307	3.57(3.16, 4.02)	3.48 (493.65)	1.51	2.92
Disturbance in attention	Nervous system disorders	306	7.22(6.37, 8.18)	7.00 (1297.36)	2.36	5.33
Agitation	Psychiatric disorders	302	3.55(3.15, 4.01)	3.46 (482.60)	1.50	2.91
Application site pruritus	General disorders and administration site conditions	211	74.91(58.59, 95.79)	73.10 (4560.75)	4.11	18.64
Psychomotor hyperactivity	Nervous system disorders	200	5.53(4.75, 6.44)	5.43 (619.90)	2.01	4.21
Tic	Psychiatric disorders	187	5.38(4.61, 6.29)	5.29 (560.37)	1.97	4.10
Irritability	Psychiatric disorders	181	3.06(2.63, 3.57)	3.02 (224.88)	1.27	2.50
Application site irritation	General disorders and administration site conditions	146	80.22(59.24, 108.62)	78.87 (3232.93)	4.03	18.17
Anger	Psychiatric disorders	140	2.51(2.11, 2.99)	2.49 (116.29)	0.98	2.06
Hypertension	Vascular disorders	137	2.46(2.07, 2.93)	2.44 (108.83)	0.95	2.02
Feeling abnormal	General disorders and administration site conditions	135	2.49(2.09, 2.98)	2.47 (110.49)	0.97	2.04
Dyskinesia	Nervous system disorders	128	3.71(3.08, 4.46)	3.67 (223.49)	1.47	2.91
Application site pain	General disorders and administration site conditions	119	11.26(9.13, 13.89)	11.12 (814.01)	2.70	7.13
Dystonia*	Nervous system disorders	111	3.49(2.87, 4.25)	3.46 (175.98)	1.37	2.73
Disturbance in social behavior*	Psychiatric disorders	110	49.31(36.61, 66.43)	48.69 (2034.15)	3.73	15.48
Hallucination, visual	Psychiatric disorders	101	4.76(3.86, 5.87)	4.71 (258.11)	1.73	3.55

*: not included in drug labels.

**Table 3:** Top 20 PTs of methylphenidate based on the ROR values.

PT	SOC	Case reports	ROR(95%CI)	PRR(χ^2^)	IC025	EBGM05
Coronary artery dissection*	Cardiac disorders	24	767.07(103.76-5670.98)	764.93(732.48)	2.99	5.92
Dermal absorption impaired*	Skin and subcutaneous tissue disorders	6	191.37(23.04-1589.76)	191.23(162.21)	1.00	4.79
Huntington’s disease*	Congenital, familial and genetic disorders	4	127.55(14.25-1141.32)	127.49(100.40)	0.33	4.20
Application site erosion	General disorders and administration site conditions	17	108.58(40.05-294.37)	108.37(411.03)	2.50	11.03
Application site erythema	General disorders and administration site conditions	418	106.85(87.70-130.18)	101.70(9964.49)	4.37	21.22
Application site oedema	General disorders and administration site conditions	9	95.72(25.91-353.62)	95.62(210.67)	1.63	8.26
Application site urticaria	General disorders and administration site conditions	26	92.35(43.26-197.14)	92.08(602.37)	2.95	12.95
Application site irritation	General disorders and administration site conditions	146	80.22(59.24-108.62)	78.87(3232.93)	4.03	18.17
Application site pruritus	General disorders and administration site conditions	211	74.91(58.59-95.79)	73.10(4560.75)	4.11	18.64
Application site papules	General disorders and administration site conditions	4	63.77(11.68-348.24)	63.74(82.35)	0.35	5.30
Application site hemorrhage	General disorders and administration site conditions	10	53.18(19.32-146.36)	53.12(191.78)	1.74	8.81
Disturbance in social behavior*	Psychiatric disorders	110	49.31(36.61-66.43)	48.69(2034.15)	3.73	15.48
Application site rash	General disorders and administration site conditions	76	46.99(33.00-66.91)	46.58(1377.77)	3.55	14.52
Polydipsia psychogenic*	Psychiatric disorders	7	44.66(14.17-140.73)	44.62(124.38)	1.22	7.34
Nail picking*	Psychiatric disorders	4	42.52(9.51-190.00)	42.50(69.46)	0.36	5.37
Application site dryness	General disorders and administration site conditions	18	33.82(17.42-65.64)	33.75(277.85)	2.36	9.71
Application site inflammation	General disorders and administration site conditions	5	31.89(9.23-110.18)	31.87(74.76)	0.70	5.82
Application site scab	General disorders and administration site conditions	7	27.91(10.12-76.99)	27.89(96.79)	1.18	6.56
Benign rolandic epilepsy*	Nervous system disorders	6	27.34(9.19-81.36)	27.32(81.92)	0.96	6.09
Application site swelling	General disorders and administration site conditions	36	25.60(16.51-39.70)	25.50(470.88)	2.83	10.12

*: not included in drug labels.

Positive signals were categorized and summarized by SOC, as detailed in **Table 4**. Overall, the top five SOCs in terms of the number of signals were psychiatric disorders (51 signals), general disorders and administration site conditions (25 signals), nervous system disorders (12 signals), cardiac disorders (9 signals), metabolism and nutrition disorders (6 signals), and vascular disorders (6 signals).

**Table 4:** Total SOC distribution and subgroup SOC distribution of ADR signals from methylphenidate.

SOC	Total		Male		Female		Oral immediate-release		Oral extended-release		Transdermal extended-release	
	signals,n	Proportion,%	signals,n	Proportion,%	signals,n	Proportion,%	signals,n	Proportion,%	signals,n	Proportion,%	signals,n	Proportion,%
Psychiatric disorders	51	38.64%	29	33.72%	38	42.22%	18	41.86%	40	45.45%	5	14.71%
General disorders and administration site conditions	25	18.94%	18	20.93%	21	23.33%	3	6.98%	3	3.41%	22	64.71%
Nervous system disorders	12	9.09%	8	9.30%	8	8.89%	7	16.28%	6	6.82%	2	5.88%
Cardiac disorders	9	6.82%	9	10.47%	5	5.56%	1	2.33%	8	9.09%	0	0.00%
Metabolism and nutrition disorders	6	4.55%	3	3.49%	2	2.22%	1	2.33%	3	3.41%	3	8.82%
Vascular disorders	6	4.55%	3	3.49%	2	2.22%	0	0.00%	2	2.27%	0	0.00%
Reproductive system and breast disorders	3	2.27%	2	2.33%	0	0.00%	0	0.00%	3	3.41%	0	0.00%
Skin and subcutaneous tissue disorders	3	2.27%	2	2.33%	1	1.11%	0	0.00%	2	2.27%	2	5.88%
Congenital, familial and genetic disorders	3	2.27%	2	2.33%	1	1.11%	1	2.33%	2	2.27%	0	0.00%
Musculoskeletal and connective tissue disorders	3	2.27%	2	2.33%	3	3.33%	1	2.33%	2	2.27%	0	0.00%
Eye disorders	3	2.27%	3	3.49%	2	2.22%	2	4.65%	5	5.68%	0	0.00%
Gastrointestinal disorders	3	2.27%	3	3.49%	0	0.00%	0	0.00%	2	2.27%	0	0.00%
Endocrine disorders	2	1.52%	1	1.16%	1	1.11%	0	0.00%	3	3.41%	0	0.00%
Injury, poisoning and procedural complications	1	0.76%	0	0.00%	0	0.00%	1	2.33%	1	1.14%	0	0.00%
Investigations	1	0.76%	0	0.00%	3	3.33%	7	16.28%	3	3.41%	0	0.00%
Respiratory, thoracic and mediastinal disorders	1	0.76%	1	1.16%	1	1.11%	1	2.33%	2	2.27%	0	0.00%
Infections and infestations	0	0.00%	0	0.00%	0	0.00%	0	0.00%	1	1.14%	0	0.00%
Neoplasms benign, malignant and unspecified (incl cysts and polyps)	0	0.00%	0	0.00%	1	1.11%	0	0.00%	0	0.00%	0	0.00%
Blood and lymphatic system disorders	0	0.00%	0	0.00%	1	1.11%	0	0.00%	0	0.00%	0	0.00%
Total	132	100.00%	86	100.00%	90	100.00%	43	100.00%	88	100.00%	34	100.00%

### 3.3 Gender differences

**Figure 3:**
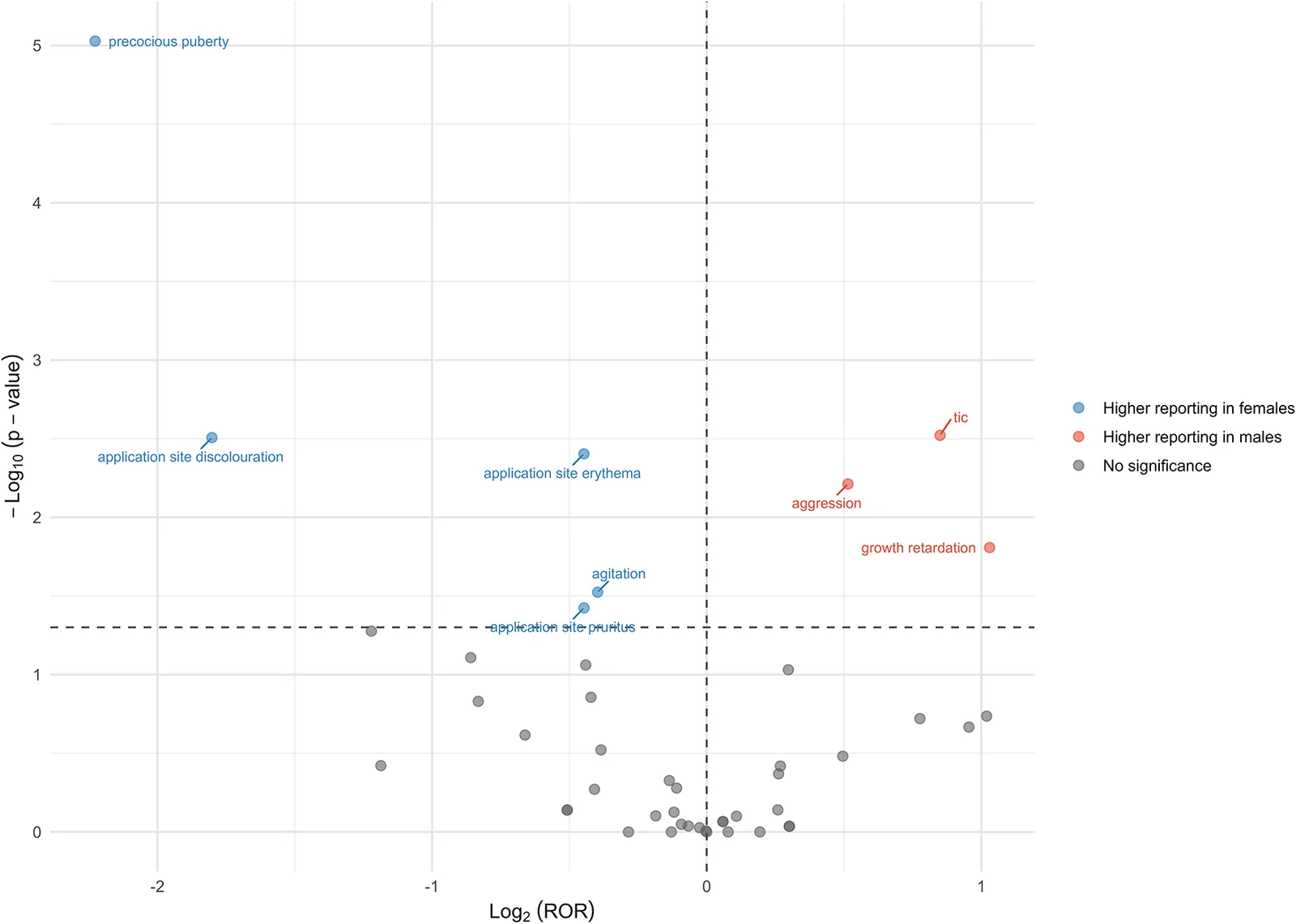
Volcano plot of gender differences in ADR signals related to methylphenidate. The vertical axis represents −log_10_(*p*-value), and the horizontal axis represents log_2_(ROR). Red dots indicate significantly higher reporting in males; blue dots indicate higher reporting in females (*p* < 0.05). The plot displays signals of disproportionate reporting, not absolute risk or causal associations.

Across the four signal detection algorithms, 86 and 90 positive ADR signals were identified in pediatric male and female patients, respectively (**Supplementary Tables 7**, **S8**). The gender-stratified distribution of methylphenidate-associated positive ADR signals across SOCs is also presented in **Table 4**. Psychiatric disorders were the most frequently affected SOC in both genders, accounting for 33.72% of male signals and 42.22% of female signals. Other differences were observed: cardiac disorders had a higher proportion of signals in males (10.47%) than in females (5.56%), whereas females had higher proportions of signals in general disorders and administration site conditions (23.33% vs. 20.93%) and in investigations (3.33% vs. 0%). Forty-one ADR signals were detected exclusively in males and 45 exclusively in females, suggesting potential gender-specific pharmacovigilance patterns (**Table 5**). Male-exclusive signals included psychiatric disorders (e.g., dysphemia, impulse-control disorder, and illusion), cardiac disorders (e.g., arrhythmia, coronary artery dissection, and acute myocardial infarction), and priapism. Female-exclusive signals included psychiatric disorders (e.g., irritability, hallucination, and mood swings), tachycardia, and application site reactions. In addition, a comparative analysis was conducted on signals detected in both genders. The analysis showed significant gender-associated divergence in signal reporting frequencies: aggression, tic, and growth retardation showed significantly higher reporting among male pediatric patients, while application site erythema, agitation, application site pruritus, precocious puberty, and application site discolouration showed significantly higher reporting among female pediatric patients (**Table 6**). A volcano plot was constructed to visually illustrate gender differences in methylphenidate-related positive signals among pediatric populations (**Figure 3**).

**Table 5:** Gender-specific ADR signals associated with methylphenidate.

SOC	Male-specific PT (case reports)	Female-specific PT (case reports)
Psychiatric disorders	dysphemia (18), impulse-control disorder (10), illusion (8), psychotic symptom (7), obsessive-compulsive symptom (7), acute psychosis (7), alice in wonderland syndrome (6), daydreaming (6), social anxiety disorder (5), disruptive mood dysregulation disorder (4), selective mutism (4)	irritability (49), hallucination (38), depressed mood (31), mood swings (31), apathy (28), anger (27), affect lability(19), emotional disorder (18), mania (16), hallucinations, mixed (10), Delusion (10), thinking abnormal (9), bruxism (8), initial insomnia (8), social avoidant behavior (8), frustration tolerance decreased (5), morose (4), compulsions (4), dysphoria (4), regressive behavior (3)
General disorders and administration site conditions	withdrawal syndrome (48), application site scab (7), application site oedema (6)	crying (36), feeling jittery (12), application site erosion (7), energy increased (4), application site dermatitis (3), application site warmth (3)
Cardiac disorders	arrhythmia (38), coronary artery dissection (24), acute myocardial infarction (18), myocardial infarction (15), mitral valve disease (5), Wolff-Parkinson-White syndrome (5), mitral valve prolapse (4)	tachycardia (73), palpitations (28), aortic valve incompetence (4)
Investigations	–	heart rate increased (36), body temperature increased (24), blood pressure increased (18)
Nervous system disorders	formication (20), oromandibular dystonia (18), benign rolandic epilepsy (6)	dystonia (28), reversible cerebral vasoconstriction syndrome (5), dyslalia (4)
Eye disorders	visual acuity reduced (15), excessive eye blinking (14), accommodation disorder (4)	mydriasis (41), visual snow syndrome (3)
Blood and lymphatic system disorders	–	immune thrombocytopenia (5)
Congenital, familial and genetic disorders	tourette’s disorder (22), gilbert’s syndrome (6)	hypertrophic cardiomyopathy (3)
Metabolism and nutrition disorders	abnormal loss of weight (7), water intoxication (4)	weight gain poor (4)
Musculoskeletal and connective tissue disorders	–	muscle contracture (12)
Neoplasms benign, malignant and unspecified (incl cysts and polyps)	–	ewing’s sarcoma (4)
Vascular disorders	peripheral vascular disorder (5), vasospasm (4)	hypertension (50)
Skin and subcutaneous tissue disorders	vitiligo (8), dermal absorption impaired (5)	livedo reticularis (4)
Respiratory, thoracic and mediastinal disorders	pulmonary hypertension (17)	tachypnea (24)
Gastrointestinal disorders	coeliac disease (13), gastrointestinal pain (8), tongue spasm (5)	–
Reproductive system and breast disorders	priapism (31), painful erection (6)	–
Total(signals,n)	41	45

**Table 6:** Gender differences in signals common to both males and females. .

PT	SOC	a	c	ROR	95%CI	*P* value	Direction
Aggression	Psychiatric disorders	304	76	1.43	1.11-1.85	0.0061	Male
Application site erythema	General disorders and administration site conditions	284	134	0.73	0.59-0.91	0.0039	Female
Agitation	Psychiatric disorders	205	94	0.76	0.59-0.97	0.0299	Female
Tic	Psychiatric disorders	152	30	1.80	1.21-2.67	0.0030	Male
Application site pruritus	General disorders and administration site conditions	143	68	0.73	0.55-0.98	0.0376	Female
Growth retardation	Musculoskeletal and connective tissue disorders	75	13	2.04	1.13-3.69	0.0156	Male
Precocious puberty	Endocrine disorders	11	18	0.21	0.10-0.45	< 0.0001	Female
Application site discolouration	General disorders and administration site conditions	9	11	0.29	0.12-0.69	0.0031	Female

“a” denotes male targeted PTs and “c” denotes female targeted PTs.

### 3.4 Dosage-form differences

**Figure 4:**
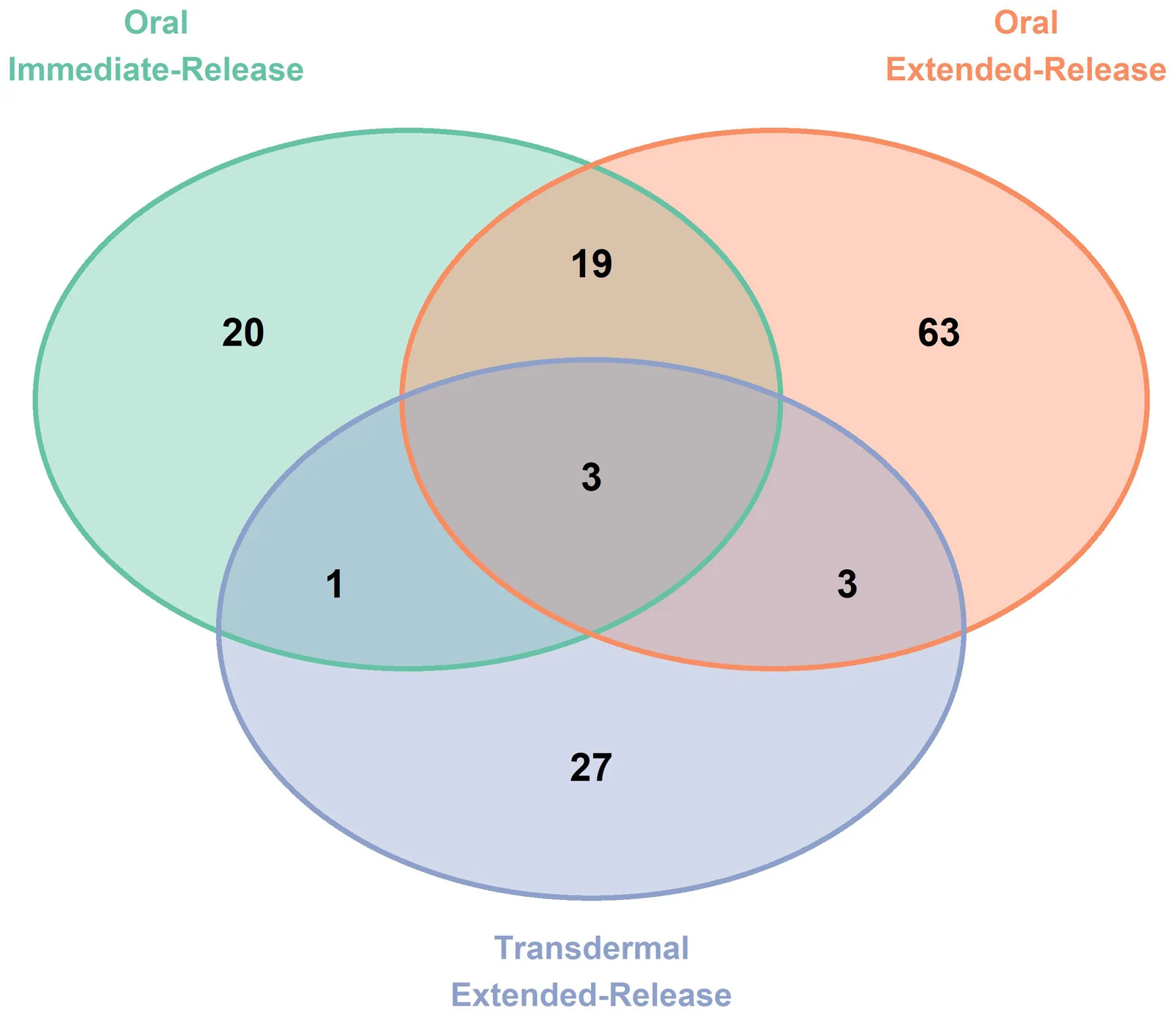
Venn diagram of positive ADR signals across three methylphenidate dosage-forms.

A total of 489 ADE reports for oral immediate-release formulations, 2,111 for oral extended-release formulations, and 2,081 for transdermal extended-release formulations were extracted. Using four disproportionality analysis methods (ROR, PRR, BCPNN, and MGPS), 43 positive signals were detected for oral immediate-release formulations, 88 for oral extended-release formulations, and 34 for transdermal extended-release formulations (**Supplementary Tables 9–S11**). The distribution of positive signals by SOC for each dosage form is detailed in **Table 4**. For oral immediate-release and oral extended-release formulations, positive signals were mainly distributed in psychiatric disorders (41.86% and 45.45%, respectively), while for the transdermal formulation, positive signals were mainly distributed in general disorders and administration site conditions (64.71%). The overlap and differences among positive signals across the three dosage forms are shown in the Venn diagram (**Figure 4**) and detailed in **Supplementary Table 12**. Only three signals were shared by all three formulations: disturbance in attention, abnormal behavior, and decreased appetite. The oral immediate-release formulation was uniquely associated with 20 signals, some of which were acute and severe (e.g., sudden death, syncope). The oral extended-release formulation exhibited the largest number of unique signals (n = 63), covering a broad spectrum of ADEs (e.g., irritability, precocious puberty, priapism, arrhythmia, suicidal ideation). The transdermal extended-release formulation was associated with 27 unique signals, nearly all of which were application site reactions (e.g., erythema, pruritus, erosion, discolouration, vesicles). Overall, the three formulations exhibited limited overlap and distinct safety profiles. The transdermal extended-release formulation was primarily associated with application site reactions, whereas the oral extended-release formulation showed the widest range of systemic signals.

### 3.5 Sensitivity analysis

In the sensitivity analysis using Bonferroni correction (N = 473), adjusted *P*-values were calculated as *P*_adj = *P*_raw × 473. Following this correction, 461 of the 473 positive signals (97.5%) remained statistically significant, including key signals such as coronary artery dissection (**Supplementary Tables 5, S7–S11**). This supports the robustness of our primary signals to multiple comparisons.

## 4 Discussion

Methylphenidate-associated ADE reports showed a marked male predominance among pediatric patients, with male reports outnumbering female reports by approximately threefold. This pattern may reflect the higher clinical diagnosis rate of ADHD in males, as well as the greater proportion of male patients receiving methylphenidate treatment (31–33). The higher volume of ADE reports in the 6–11-year-old age group compared with adolescents (12–17 years) is consistent with population-based prescribing data showing higher ADHD diagnosis and methylphenidate treatment rates in younger school-aged children, as reported in a nationwide French cohort study (13).

### 4.1 Overall safety signals

In this study, ADEs associated with methylphenidate predominantly affected the psychiatric system, consistent with the SOC distribution and with global case reports of pediatric ADEs related to methylphenidate use (34). Psychiatric ADR signals primarily included aggression, insomnia, agitation, tic, irritability, and anger, which are well documented in the literature (35, 36) and drug labels (9). The neurobiological basis of these psychiatric effects is thought to involve excessive dopaminergic signaling in the striatum of susceptible individuals (37). By enhancing dopaminergic transmission in the mesolimbic and mesocortical pathways, methylphenidate may precipitate or exacerbate psychiatric symptoms, particularly in those with preexisting vulnerability (38). Case reports have documented symptom remission following drug discontinuation (39). Although tics may present as comorbid conditions in individuals with ADHD, the occurrence of sudden-onset, persistent, or particularly severe tics should prompt clinicians to consider secondary etiologies, including the use of dopaminergic agents such as methylphenidate (10). In addition, a substantial number of administration site reaction signals were identified, which were primarily associated with the transdermal delivery system of methylphenidate. The most commonly observed reaction was application site erythema. This erythema usually causes mild or no discomfort and generally does not interfere with treatment or necessitate treatment discontinuation. However, if erythema is accompanied by severe local reactions, such as edema or blister formation, and fails to show significant improvement within 48 hours or extends beyond the application site of the transdermal system, contact sensitization should be suspected, and the patch should be discontinued (40).

Several ADR signals not previously documented in drug labels were identified. Most pertain to psychiatric disorders (e.g., abnormal behavior, disturbance in social behavior), which may be confounded by underlying ADHD or comorbid conditions (41). The core symptoms of ADHD (hyperactivity, impulsivity, and inattention) and comorbid psychiatric disorders frequently manifest as behavioral disturbances (41–43). This overlap renders it inherently challenging to distinguish potential drug effects from disease-related manifestations in SRS. Therefore, we interpret these psychiatric signals as hypothesis-generating and caution against causal inference. Future pharmacoepidemiological studies incorporating longitudinal data and baseline symptom assessments would be valuable to clarify the contributions of drug exposure versus underlying disease to these observed associations. Coronary artery dissection was identified as a novel and potentially important ADR signal in this study. Of the 24 cases, 23 had no concomitant medications, virtually excluding drug interactions as a confounder. The remaining case reported five “Interacting” drugs, which are more consistent with post-myocardial infarction therapy than with suspected etiologic agents. A case report described a 6-year-old boy with ADHD who experienced acute myocardial infarction caused by spontaneous coronary artery dissection on the third day following methylphenidate administration (44). Additional cases of coronary artery dissection (45) and myocardial infarction (46, 47) have also been reported in adults. From a pharmacological perspective, methylphenidate exerts sympathomimetic effects, including elevations in heart rate, blood pressure, and myocardial contractility (48). Such hemodynamic changes could, in theory, increase mechanical stress on the coronary arterial wall and predispose susceptible individuals to intimal injury. Epidemiological studies have reported associations between methylphenidate and cardiovascular conditions including hypertension, arterial disease, and arrhythmias, although associations with stroke, myocardial infarction, or all-cause mortality were not statistically significant (49, 50). Notably, ADHD itself may act as an independent cardiovascular risk factor (51), potentially confounding these observations. Given the exploratory nature of FAERS-based signal detection, the coronary artery dissection signal should be viewed as hypothesis-generating rather than confirmatory. A causal relationship cannot be established from this study; further pharmacoepidemiological studies are needed to confirm or refute this signal. From a clinical standpoint, clinicians should remain vigilant and consider cardiovascular evaluation in pediatric patients presenting with acute chest pain, syncope, or unexplained cardiorespiratory symptoms, particularly those with pre-existing cardiovascular risk factors. An ADR signal for Huntington’s disease was also identified. Given its established genetic etiology, this signal is unlikely to represent a genuine ADR. This signal more likely reflects protopathic bias, wherein methylphenidate was prescribed for early motor or behavioral symptoms of an undiagnosed genetic disorder, and the underlying disease was subsequently reported as an ADE. Therefore, this association should not be interpreted as causal.

### 4.2 Gender-specific patterns

In this study, we identified 86 positive ADR signals in males and 90 in females. Among these, 41 were male-exclusive, 45 female-exclusive, and 45 were detected in both genders. These exclusive signals should be interpreted with caution, as they may arise from multiple comparisons, low statistical power, or differences in exposure prevalence rather than true biological specificity. They should therefore be considered hypothesis-generating rather than definitive evidence. Cardiovascular ADR signals showed marked gender differences in signal composition and severity. Male-exclusive signals comprised severe, life-threatening events including coronary artery dissection, acute myocardial infarction, myocardial infarction, and Wolff-Parkinson-White syndrome, whereas female patients predominantly reported tachycardia and palpitations. In a series of case reports (44–47), all patients who developed coronary artery dissection or acute myocardial infarction following methylphenidate use were male, including one boy. This male predominance may be partly explained by the relative lack of estrogen-mediated vascular protection in males. Estrogen has been shown to counteract cardiac remodeling and regulate vascular tone (52–54), and its absence could theoretically reduce vascular resilience to acute hemodynamic stress. Tachycardia and palpitations are predominantly reported in female patients, likely reflecting a combination of enhanced dopaminergic sensitivity to methylphenidate and/or a lower threshold for reporting subjective symptoms (55–57). These findings underscore the necessity of gender-specific monitoring and further research to validate causal relationships and disentangle biological from non-biological contributors.

Among signals identified in both genders, several showed statistically significant gender differences. Boys had significantly higher reporting of aggression, tic, and growth retardation. The male predominance in tic reporting aligns with the higher baseline prevalence of tic disorders in males (58). In a retrospective study of 121 children with ADHD, tic aggravation occurred exclusively in male patients (10/100 males vs. 0/21 females), with Cox regression identifying male gender as a significant risk factor (*p* < 0.001) (59). However, the retrospective design precluded establishing a causal link. Clinicians should remain vigilant to potential tic development in male pediatric patients receiving methylphenidate. Growth retardation is a recognized ADR of methylphenidate (9). In our study, the reporting frequency of growth retardation was significantly higher in boys than in girls. However, longitudinal studies (60, 61) have suggested that long-term methylphenidate use has little to no effect on childhood growth, and any observed impact is slight and typically resolves by adulthood. A 24-month longitudinal study found no gender differences in changes in height, weight, or body mass index (62). The male predominance in growth retardation reporting may partly reflect the higher prescribing volume in males (32, 33), rather than a genuine gender difference. In contrast, girls had significantly higher reporting of application site erythema, application site pruritus, application site discolouration, precocious puberty and agitation. The female predominance of application site reactions may be partly attributable to either differences in skin sensitivity or gender-based disparities in ADE reporting (56, 57). Regarding precocious puberty, although female patients showed higher reporting than males in our study, this observation should be interpreted with caution. The substantially higher baseline prevalence in girls (63) and the increased risk of precocious puberty in children with ADHD, regardless of medication use (64, 65), suggest that the evidence for a causal link remains inconclusive. Nonetheless, clinicians should remain vigilant to this potential adverse outcome, especially when treating girls with ADHD. Aggression and agitation showed opposite gender patterns in this study. The higher reporting of aggression in males and agitation in females likely reflects a combination of gender-specific behavioral expression, potential neurobiological differences, and differential symptom recognition/reporting bias inherent to FAERS (55, 66). Although these statistically significant gender differences require further validation through subsequent research, they provide valuable insights for enhancing gender-specific pharmacovigilance strategies in pediatric populations.

### 4.3 Dosage-form-specific patterns

The three dosage forms exhibited distinct ADR signal profiles, likely reflecting their different routes of administration and pharmacokinetic properties (67). For the oral immediate-release and oral extended-release formulations, psychiatric disorders were the most frequently reported SOC (41.86% and 45.45% of signals, respectively). This aligns with the known central nervous system stimulant profile of methylphenidate, which is commonly associated with insomnia, agitation, and aggression (9, 36). In contrast, for the transdermal extended-release formulation, 64.71% of ADR signals were classified under “General disorders and administration site conditions”. This distribution is consistent with the local tolerability profile of transdermal delivery: the FDA-approved labeling lists a broad spectrum of application site reactions, including erythema, pruritus, hyperpigmentation, erosion, and vesicles (68). Clinical trial data indicate that mild-to-moderate erythema occurs in approximately 50% of users, while allergic contact dermatitis remains uncommon (<1%) (69). Only three PTs were shared across all three formulations: disturbance in attention, abnormal behavior, and decreased appetite. These may reflect the core systemic ADR profile of methylphenidate, the inherent symptoms of ADHD or comorbidity, or a combination of both. Decreased appetite is a well-documented, dose-dependent effect of methylphenidate, mediated by dopaminergic and noradrenergic pathways that regulate appetite (36). In pediatric patients, decreased appetite may be associated with compromised growth trajectories, necessitating long-term, close monitoring. The oral immediate-release formulation contributed 20 unique signals, including acute severe ADR signals such as sudden death (n = 10) and syncope (n = 12). These serious cardiovascular ADRs have been explicitly specified in the drug label. A meta-analysis (50) and a case-control study (70) reported positive associations between methylphenidate use in children/adolescents and sudden death or arrhythmia, though the absolute risk is low. A nationwide self-controlled case series study also reported elevated relative risks of myocardial infarction and arrhythmias after methylphenidate initiation, although it did not perform a formulation-specific analysis (71). The exclusive identification of these signals with oral immediate-release formulations is likely explained by their pharmacokinetic profile of rapid peak plasma concentrations (72), which may trigger abrupt hemodynamic stress and precipitate arrhythmias in susceptible individuals. Oral extended-release formulations accounted for the largest number of unique signals (n = 63), spanning psychiatric, cardiovascular, and endocrine events. While this may partly reflect higher prescribing volume (73), the cardiovascular signals identified—including supraventricular tachycardia, extrasystoles, angina pectoris, chest pain, and palpitations—are consistent with the sympathomimetic effects of methylphenidate, which increase heart rate, myocardial contractility, and peripheral vascular resistance (48). The prolonged action of these formulations may sustain these effects over hours (36), supporting clinical monitoring of blood pressure, heart rate, and related symptoms. Transdermal extended-release formulations contributed 27 unique signals, almost related to application site reactions, suggesting that local skin effects are the dominant safety concern for the patch (74). In 2015, the FDA issued a warning (75) indicating that the application of transdermal methylphenidate systems may be associated with permanent skin discoloration, called chemical leukoderma. Patients and caregivers should be advised to closely monitor for skin discoloration and report such findings promptly. Weight-related signals showed dosage-form-specific patterns: immediate-release formulations manifested as “weight decreased,” while extended-release formulations presented as “underweight” or “abnormal loss of weight.” In 2025, the FDA warned that extended-release stimulants for ADHD are associated with an elevated risk of clinically significant weight loss in children under 6 years of age due to higher plasma exposure at equivalent doses (76). This regulatory update indirectly supports our finding that extended-release formulations may confer a greater cumulative weight-related risk than immediate-release formulations. However, as with all FAERS-based signals, these findings are hypothesis-generating and require validation in pharmacoepidemiological studies with adequate exposure data.

Collectively, these findings may support formulation-specific safety monitoring. However, given the absence of exposure denominators in FAERS, our observed signal patterns are exploratory and hypothesis-generating. To move beyond signal detection toward causal inference, further validation requires approaches that provide reliable person-time at risk and adjust for confounding by indication. These include new-user active-comparator cohort designs and pharmacokinetic/pharmacodynamic modeling informed by real-world treatment duration and dose-titration data (77, 78).

### 4.4 Limitations

This study has several limitations. First, data cleaning and processing were performed using OpenVigil 2.1, which retained only reports with complete and unambiguously identifiable drug names (79, 80). Because raw data of excluded reports are inaccessible, direct comparison of key variables (e.g., age, gender, dosage form) between included and excluded reports was not possible. Although exclusion was based on the completeness and mapping of drug names, a technical criterion independent of demographics or dosage form, the possibility of selection bias cannot be fully excluded. The platform also provided only 17 predefined fields and did not include critical information such as exact treatment duration or the precise timing of ADEs. The absence of these variables precluded in-depth time-to-event analyses. These limitations should be considered when interpreting the findings. Second, disproportionality analyses based on SRS are inherently limited by data quality. SRS data are subject to underreporting, selective reporting, and duplicate reporting. The absence of denominator data means that observed differences in signal counts across strata cannot be interpreted as differences in incidence rates, relative risks, or causal effects. They may partly reflect variations in prescribing patterns, exposure prevalence, and reporting behaviors. Our stratified findings should therefore be viewed exclusively as exploratory and hypothesis-generating, requiring confirmation through population-based pharmacoepidemiological studies with complete exposure data. Third, this study included only reports in which methylphenidate was designated as the PS drug and did not assess potential drug–drug interactions or ADR signals associated with concomitant medications. Age-stratified analyses were also not performed due to the absence of a clear pharmacokinetic breakpoint within the 6–17-year age range (81). Future studies with larger samples across broader age groups are warranted to explore age-specific differences. Fourth, to enhance specificity and minimize false positives, we adopted a composite rule requiring simultaneous positivity across all four disproportionality algorithms (ROR, PRR, BCPNN, and MGPS). This intentionally conservative approach may miss genuine but weak associations, particularly when Bayesian shrinkage strongly attenuates estimates for rare events (IC025 ≤ 0 or EBGM05 ≤ 2). Absence of a disproportionality signal should therefore not be equated with absence of risk. Finally, inherent limitations exist when applying disproportionality analysis to SRS data. Findings from such analyses cannot establish causal relationships but may serve as a basis for hypothesis generation. The observed associations require further validation through prospective clinical studies.

## 5 Conclusion

Due to inherent limitations of SRS data, including incomplete case ascertainment, reporting biases, and lack of denominator information, causal inference cannot be established from this FAERS-based analysis. All signals identified should therefore be interpreted strictly as hypothesis-generating. In this analysis, we detected overall, gender-stratified, and dosage-form-specific ADR signals for methylphenidate in pediatric patients, including a coronary artery dissection signal not currently listed in drug labels. Gender-specific patterns were observed: boys showed a higher reporting frequency of serious cardiac events, whereas girls showed a higher frequency of application-site reactions. Dosage-form-specific patterns revealed that acute severe events predominated with the immediate-release formulation; the oral extended-release formulation accounted for the greatest number of signals; and the transdermal patch was predominantly associated with local cutaneous reactions. These findings support dosage-form- and gender-informed pharmacovigilance. Well-designed prospective studies are needed to rigorously evaluate the clinical relevance and potential causal associations underlying these signals.

## Data Availability

The original contributions presented in the study are included in the article/**Supplementary Material**. Further inquiries can be directed to the corresponding author.
