# Supplementary material for: Disproportionality analysis of methylphenidate-associated adverse events in pediatric patients using the FDA Adverse Event Reporting System: overall signals and stratification by gender and dosage form
Source: Front Psychiatry. 2026 Aug 12;17:1896696. doi: 10.3389/fpsyt.2026.1896696 (PMC13506775; doi:10.3389/fpsyt.2026.1896696)
Supplement: Supplementary file 1 [file Table1.docx]

Supplementary Material

**Supplementary Table S1. Summary of data extraction, processing, and analytical procedures.**

| Steps | Operation | Counts |
| --- | --- | --- |
| Data extraction and filtering | OpenVigil 2.1 platform  **Overall**  - Drug: methylphenidate  - Role of drug: Primary Suspect  - Age of patient: 6–17  - Report submitted: 2004Q1-2025Q1  - Evaluation method: Raw_data; Frequentist_methods  - Counting records according to: ISR (most recent)  - Output format of query result: CSV  **Gender subgroups**  For the “Patient Gender” field, select “Male” or “Female.” All other fields match the corresponding entries in the overall signal analysis.  **Dosage-form subgroups**  - Oral immediate-release formulations: Pharmaproduct = methylin or ritalin  - Oral extended-release formulations: Pharmaproduct = adhansia xr or aptensio xr or biphentin or cotempla xr-odt or equasym xl or foquest or jornay pm extended-release or metadate or metadate cd or metadate er or methylin er or quillichew er or quillivant xr or ritalin la or ritalin sr or ritalin-sr or concerta  - Transdermal extended-release formulations: Pharmaproduct = daytrana  All other fields match the corresponding entries in the overall signal analysis. | **Overall**  - 8585 reports (Primary Suspect)  - 1945 adverse events  **Male subgroup**  - 6210 reports (Primary Suspect)  - 1687 adverse events  **Female subgroup**  - 2185 reports (Primary Suspect)  - 977 adverse events  **Oral immediate-release formulation subgroup**  - 489 reports (Primary Suspect)  - 654 adverse events  **Oral extended-release formulation subgroup**  - 2111 reports (Primary Suspect)  - 1140 adverse events  **Transdermal extended-release formulation subgroup**  - 2081 reports (Primary Suspect)  - 384 adverse events |
| Data processing | MedDRA 27.0 PT Mapping | - **Overall:** 1843 PTs  - **Gender subgroups:** 1599 PTs (Male); 926 PTs (Female)  - **Dosage-form subgroups:** 637 PTs (Oral immediate-release formulation); 1083 PTs (Oral extended-release formulation); 373 PTs (Transdermal extended-release formulation) |
| Statistical analysis | R 4.5.0  - Ovrall analysis (ROR, PRR, BCPNN, MGPS)  - Gender subgroups (ROR, PRR, BCPNN, MGPS)  - Gender differences in shared signals (ROR)  - Dosage-form subgroups (ROR, PRR, BCPNN, MGPS)  - Sensitivity analysis (Bonferroni correction) | - **Overall: 132 PTs** (172 PTs meet the criteria of the four algorithms, followed by the exclusion of 40 PTs: attention deficit hyperactivity disorder, incorrect product administration duration, no adverse event, product administered at inappropriate site, product administration error, product complaint, product prescribing error, product residue present, therapeutic product effect decreased, therapeutic product effect delayed, therapeutic product effect increased, therapeutic product effect prolonged, therapeutic product effect variable, wrong technique in product usage process, product quality issue, product substitution issue, product adhesion issue, educational problem, device adhesion issue, device breakage, adhesive tape use, product availability issue, therapeutic response changed, therapeutic product ineffective, product taste abnormal, product formulation issue, device difficult to use, product colour issue, rebound effect, gastric lavage, incorrect dose administered by product, drug screen negative, drug diversion, fight in school, heat stroke, product solubility abnormal, drug delivery system issue, product coating issue, drug tolerance, exposure via direct contact)  - **Male: 86 PTs** (119 PTs meet the criteria of the four algorithms, followed by the exclusion of 33 PTs: product quality issue, product substitution issue, product adhesion issue, device adhesion issue, device breakage, product taste abnormal, product formulation issue, product colour issue, product coating issue, adhesive tape use, resuscitation, incorrect product administration duration, product administered at inappropriate site, product administration error, product prescribing error, wrong technique in product usage process, device difficult to use, exposure via direct contact, heat stroke, product residue present, drug screen negative, no adverse event, therapeutic product effect decreased, therapeutic product effect incomplete, therapeutic product effect increased, therapeutic product effect delayed, therapeutic product ineffective, therapeutic response changed, drug tolerance, educational problem, drug diversion, fight in school, attention deficit hyperactivity disorder)  - **Female: 90 PTs** (118PTs meet the criteria of the four algorithms, followed by the exclusion of 28 PTs: product residue present, educational problem, product quality issue, product substitution issue, product adhesion issue, device adhesion issue, device breakage, product availability issue, product taste abnormal, product formulation issue, suspected counterfeit product, accidental exposure to product, product administered at inappropriate site, product administration error, product prescribing error, wrong technique in product usage process, incorrect product administration duration, expired product administered, device difficult to use, therapeutic product effect decreased, therapeutic product effect delayed, therapeutic response unexpected, therapeutic product effect increased, therapeutic response changed, therapeutic product ineffective, drug tolerance, no adverse event, attention deficit hyperactivity disorder)  - **Oral immediate-release formulation: 43 PTs (**52 PTs meet the criteria of the four algorithms, followed by the exclusion of 9 PTs: attention deficit hyperactivity disorder, product complaint, therapeutic product effect decreased, therapeutic response unexpected, gastric lavage, fight in school, road traffic accident, surgery, upper limb fracture**)**  - **Oral extended-release formulation: 88 PTs** (98PTs meet the criteria of the four algorithms, followed by the exclusion of 10 PTs: product residue present, therapeutic product effect decreased, product substitution issue, product availability issue, adverse event, educational problem, fight in school, loss of therapeutic response, product colour issue, contraindication to medical treatment)  - **Transdermal extended-release formulation: 34 PTs** (53 PTs meet the criteria of the four algorithms, followed by the exclusion of 19 PTs: product quality issue, product complaint, product adhesion issue, product administration error, wrong technique in product usage process, incorrect product administration duration, product administered at inappropriate site, product dose omission issue, product prescribing error, expired product administered, product storage error, therapeutic product effect delayed, therapeutic product effect decreased, therapeutic product effect increased, drug ineffective, no adverse event, educational problem, drug diversion, accidental exposure to product) |

**Supplementary Table S2. Fourfold table of disproportionality analysis.**

| Drug | Targeted PTs | The other PTs | Summation |
| --- | --- | --- | --- |
| Target drug | a | b | a+b |
| Other drugs | c | d | c+d |
| Summation | a+c | b+d | N= a+b+c+d |

**Supplementary Table S3. Four algorithms used for signal detection.**

| Algorithms | Equation | Criteria |
| --- | --- | --- |
| ROR | $ROR=\frac{\text{（}a/c\text{）}}{(b/d)}=\frac{ad}{bc}$ $95\%CI=\text{e}^{\ln\text{（}ROR\text{）}\pm1.96\sqrt{\text{（}\frac{1}{\text{a}}+\frac{1}{b}+\frac{1}{c}+\frac{1}{d}\text{）}}}$   | a≥3 with a lower 95% CI > 1 |
| PRR | $PRR=\frac{\frac{a}{(a+b)}}{\frac{c}{(c+d)}}$ $\chi^{2}=\frac{(ad-bc)^{2}(a+b+c+d)}{(a+b)(c+d)(a+c)(b+d)}$   | a≥3, PRR≥2, χ^2^≥4 |
| BCPNN | $V(IC)=\frac{1}{\text{(l}n2)^{2}}\left\{\left[\frac{(a+b+c+d-a+\gamma-\gamma11)}{(a+\gamma11)(1+a+b+c+d+\gamma)}\right]+\left[\frac{(a+b+c+d)-(a+b)+\alpha-\alpha1}{(a+b+\alpha1)(1+a+b+c+d+\alpha)}\right]+\left[\frac{(a+b+c+d)-(a+c)+\beta-\beta1}{(a+c+\beta1)(1+a+b+c+d+\beta)}\right]\right\}$ $\gamma=\gamma11\frac{(a+b+c+d+\alpha)(a+b+c+d+\beta)}{(a+b+\alpha1)(a+b+\beta1)}$ $IC-2SD=E(IC)-2\sqrt{V(IC)}$          *α=β=2, α1=β1=γ11=1* | IC025>0 |
| MGPS |  | EBGM05>2 |

Abbreviation: ROR, reporting odds ratio; PRR, proportional reporting ratio; BCPNN, Bayesian confidence propagation neural network; MGPS, multi-item gamma Poisson shrinker; EBGM, empirical Bayesian geometric mean; 95%CI, 95% confidence interval; χ^2^, chi-squared; IC, information component; IC025, the lower bound of 95% CI of the IC; EBGM05, the lower bound of 95% CI of EBGM.

**Supplementary Table S4. Fourfold table for disproportionality analysis of the gender difference in ADR signals with methylphenidate**

| Gender | Targeted PTs | The other PTs | Summation |
| --- | --- | --- | --- |
| Male | a | b | a+b |
| Female | c | d | c+d |
| Summation | a+c | b+d | N= a+b+c+d |

Note:$ROR=\frac{\text{（}a/c\text{）}}{(b/d)}=\frac{ad}{bc}$ ;$95\%CI=\text{e}^{\ln\text{（}ROR\text{）}\pm1.96\sqrt{\text{（}\frac{1}{\text{a}}+\frac{1}{b}+\frac{1}{c}+\frac{1}{d}\text{）}}}$

**Supplementary Table S5. Overall positive ADR signals associated with methylphenidate.**

| PT | SOC | Case reports | ROR | 95%CI | | PRR | χ^2^ | IC025 | EBGM05 | *P*_adj (Bonferroni) |
| --- | --- | --- | --- | --- | --- | --- | --- | --- | --- | --- |
|  |  |  |  | lower | upper |  |  |  |  |  |
| Application site erythema | General disorders and administration site conditions | 418 | 106.85 | 87.70 | 130.18 | 101.70 | 9964.49 | 4.37 | 21.22 | 0 |
| Abnormal behaviour | Psychiatric disorders | 403 | 3.94 | 3.55 | 4.38 | 3.80 | 753.73 | 1.65 | 3.21 | 2.9E-163 |
| Aggression | Psychiatric disorders | 389 | 3.34 | 3.00 | 3.72 | 3.23 | 553.48 | 1.44 | 2.77 | 1E-119 |
| Decreased appetite | Metabolism and nutrition disorders | 372 | 4.07 | 3.65 | 4.54 | 3.94 | 734.07 | 1.68 | 3.30 | 5.5E-159 |
| Insomnia | Psychiatric disorders | 307 | 3.57 | 3.16 | 4.02 | 3.48 | 493.65 | 1.51 | 2.92 | 1.1E-106 |
| Disturbance in attention | Nervous system disorders | 306 | 7.22 | 6.37 | 8.18 | 7.00 | 1297.36 | 2.36 | 5.33 | 2E-281 |
| Agitation | Psychiatric disorders | 302 | 3.55 | 3.15 | 4.01 | 3.46 | 482.60 | 1.50 | 2.91 | 2.7E-104 |
| Application site pruritus | General disorders and administration site conditions | 211 | 74.91 | 58.59 | 95.79 | 73.10 | 4560.75 | 4.11 | 18.64 | 0 |
| Psychomotor hyperactivity | Nervous system disorders | 200 | 5.53 | 4.75 | 6.44 | 5.43 | 619.90 | 2.01 | 4.21 | 3.7E-134 |
| Tic | Psychiatric disorders | 187 | 5.38 | 4.61 | 6.29 | 5.29 | 560.37 | 1.97 | 4.10 | 3.3E-121 |
| Irritability | Psychiatric disorders | 181 | 3.06 | 2.63 | 3.57 | 3.02 | 224.88 | 1.27 | 2.50 | 3.69E-48 |
| Application site irritation | General disorders and administration site conditions | 146 | 80.22 | 59.24 | 108.62 | 78.87 | 3232.93 | 4.03 | 18.17 | 0 |
| Anger | Psychiatric disorders | 140 | 2.51 | 2.11 | 2.99 | 2.49 | 116.29 | 0.98 | 2.06 | 1.95E-24 |
| Hypertension | Vascular disorders | 137 | 2.46 | 2.07 | 2.93 | 2.44 | 108.83 | 0.95 | 2.02 | 8.37E-23 |
| Feeling abnormal | General disorders and administration site conditions | 135 | 2.49 | 2.09 | 2.98 | 2.47 | 110.49 | 0.97 | 2.04 | 3.62E-23 |
| Dyskinesia | Nervous system disorders | 128 | 3.71 | 3.08 | 4.46 | 3.67 | 223.49 | 1.47 | 2.91 | 7.41E-48 |
| Application site pain | General disorders and administration site conditions | 119 | 11.26 | 9.13 | 13.89 | 11.12 | 814.01 | 2.70 | 7.13 | 2.3E-176 |
| Dystonia | Nervous system disorders | 111 | 3.49 | 2.87 | 4.25 | 3.46 | 175.98 | 1.37 | 2.73 | 1.73E-37 |
| Disturbance in social behaviour | Psychiatric disorders | 110 | 49.31 | 36.61 | 66.43 | 48.69 | 2034.15 | 3.73 | 15.48 | 0 |
| Hallucination, visual | Psychiatric disorders | 101 | 4.76 | 3.86 | 5.87 | 4.71 | 258.11 | 1.73 | 3.55 | 2.09E-55 |
| Restlessness | Psychiatric disorders | 101 | 4.21 | 3.42 | 5.18 | 4.17 | 215.88 | 1.58 | 3.19 | 3.38E-46 |
| Growth retardation | Musculoskeletal and connective tissue disorders | 94 | 4.80 | 3.86 | 5.97 | 4.76 | 243.76 | 1.73 | 3.56 | 2.82E-52 |
| Psychotic disorder | Psychiatric disorders | 91 | 3.24 | 2.61 | 4.02 | 3.21 | 126.37 | 1.24 | 2.51 | 1.21E-26 |
| Palpitations | Cardiac disorders | 82 | 2.91 | 2.32 | 3.65 | 2.89 | 93.32 | 1.09 | 2.26 | 2.1E-19 |
| Application site rash | General disorders and administration site conditions | 76 | 46.99 | 33.00 | 66.91 | 46.58 | 1377.77 | 3.55 | 14.52 | 6.7E-299 |
| Hallucination, auditory | Psychiatric disorders | 70 | 3.07 | 2.40 | 3.93 | 3.06 | 88.62 | 1.13 | 2.34 | 2.26E-18 |
| Impulsive behaviour | Psychiatric disorders | 66 | 5.80 | 4.46 | 7.55 | 5.76 | 220.37 | 1.86 | 4.04 | 3.55E-47 |
| Withdrawal syndrome | General disorders and administration site conditions | 59 | 4.20 | 3.20 | 5.52 | 4.18 | 126.36 | 1.47 | 3.03 | 1.21E-26 |
| Logorrhoea | Psychiatric disorders | 50 | 10.01 | 7.28 | 13.76 | 9.96 | 307.29 | 2.33 | 6.00 | 4.01E-66 |
| Affect lability | Psychiatric disorders | 48 | 4.16 | 3.08 | 5.63 | 4.15 | 101.54 | 1.40 | 2.94 | 3.3E-21 |
| Arrhythmia | Cardiac disorders | 47 | 2.86 | 2.12 | 3.85 | 2.85 | 51.84 | 0.94 | 2.10 | 2.85E-10 |
| Apathy | Psychiatric disorders | 46 | 4.64 | 3.41 | 6.33 | 4.62 | 114.28 | 1.51 | 3.21 | 5.35E-24 |
| Mania | Psychiatric disorders | 43 | 2.86 | 2.09 | 3.91 | 2.85 | 47.46 | 0.92 | 2.08 | 2.65E-09 |
| Tachypnoea | Respiratory, thoracic and mediastinal disorders | 42 | 2.93 | 2.13 | 4.02 | 2.92 | 48.54 | 0.94 | 2.11 | 1.53E-09 |
| Sudden death | General disorders and administration site conditions | 41 | 10.67 | 7.49 | 15.20 | 10.62 | 268.24 | 2.30 | 6.11 | 1.3E-57 |
| Paranoia | Psychiatric disorders | 40 | 3.04 | 2.20 | 4.21 | 3.04 | 49.93 | 0.98 | 2.18 | 7.54E-10 |
| Social avoidant behaviour | Psychiatric disorders | 38 | 3.22 | 2.31 | 4.50 | 3.21 | 52.68 | 1.03 | 2.28 | 1.86E-10 |
| Body temperature increased | Investigations | 38 | 3.07 | 2.20 | 4.28 | 3.06 | 48.11 | 0.97 | 2.18 | 1.91E-09 |
| Application site swelling | General disorders and administration site conditions | 36 | 25.60 | 16.51 | 39.70 | 25.50 | 470.88 | 2.83 | 10.12 | 9.8E-102 |
| Trismus | Musculoskeletal and connective tissue disorders | 31 | 6.39 | 4.35 | 9.41 | 6.37 | 117.14 | 1.71 | 3.97 | 1.27E-24 |
| Priapism | Reproductive system and breast disorders | 31 | 6.16 | 4.19 | 9.05 | 6.14 | 111.85 | 1.67 | 3.85 | 1.82E-23 |
| Precocious puberty | Endocrine disorders | 29 | 7.24 | 4.84 | 10.84 | 7.22 | 126.79 | 1.79 | 4.33 | 9.78E-27 |
| Initial insomnia | Psychiatric disorders | 29 | 3.71 | 2.52 | 5.45 | 3.70 | 51.18 | 1.10 | 2.48 | 3.98E-10 |
| Euphoric mood | Psychiatric disorders | 28 | 4.92 | 3.30 | 7.32 | 4.90 | 75.46 | 1.39 | 3.14 | 1.76E-15 |
| Hyperkinesia | Nervous system disorders | 27 | 24.66 | 14.92 | 40.76 | 24.59 | 344.97 | 2.61 | 9.40 | 2.5E-74 |
| Choreoathetosis | Nervous system disorders | 27 | 17.26 | 10.80 | 27.58 | 17.21 | 267.78 | 2.42 | 7.79 | 1.64E-57 |
| Application site urticaria | General disorders and administration site conditions | 26 | 92.35 | 43.26 | 197.14 | 92.08 | 602.37 | 2.95 | 12.95 | 2.4E-130 |
| Delusion | Psychiatric disorders | 26 | 3.21 | 2.14 | 4.80 | 3.20 | 35.76 | 0.90 | 2.14 | 1.05E-06 |
| Tourette's disorder | Congenital, familial and genetic disorders | 25 | 6.10 | 3.97 | 9.36 | 6.08 | 89.21 | 1.56 | 3.68 | 1.68E-18 |
| Coronary artery dissection | Cardiac disorders | 24 | 767.07 | 103.76 | 5670.98 | 764.93 | 732.48 | 2.99 | 5.92 | 1.2E-158 |
| Hallucinations, mixed | Psychiatric disorders | 24 | 3.99 | 2.61 | 6.11 | 3.98 | 47.72 | 1.11 | 2.56 | 2.33E-09 |
| Cardiac valve disease | Cardiac disorders | 24 | 23.97 | 14.11 | 40.71 | 23.90 | 301.03 | 2.50 | 9.04 | 9.28E-65 |
| Peripheral coldness | Vascular disorders | 23 | 3.18 | 2.07 | 4.88 | 3.17 | 31.16 | 0.84 | 2.08 | 1.12E-05 |
| Muscle contracture | Musculoskeletal and connective tissue disorders | 23 | 11.66 | 7.23 | 18.81 | 11.64 | 163.85 | 2.05 | 5.89 | 7.71E-35 |
| Feeling jittery | General disorders and administration site conditions | 22 | 3.70 | 2.38 | 5.75 | 3.69 | 38.70 | 0.99 | 2.36 | 2.33E-07 |
| Hallucination, tactile | Psychiatric disorders | 22 | 13.26 | 8.06 | 21.81 | 13.23 | 175.81 | 2.11 | 6.36 | 1.88E-37 |
| Mitral valve incompetence | Cardiac disorders | 22 | 6.22 | 3.94 | 9.82 | 6.21 | 80.44 | 1.51 | 3.65 | 1.41E-16 |
| Trichotillomania | Psychiatric disorders | 21 | 9.06 | 5.58 | 14.72 | 9.04 | 117.09 | 1.81 | 4.84 | 1.3E-24 |
| Formication | Nervous system disorders | 21 | 8.94 | 5.51 | 14.52 | 8.92 | 115.48 | 1.80 | 4.79 | 2.92E-24 |
| Dysphemia | Psychiatric disorders | 21 | 5.20 | 3.28 | 8.25 | 5.19 | 61.10 | 1.32 | 3.13 | 2.57E-12 |
| Application site discolouration | General disorders and administration site conditions | 20 | 22.03 | 12.46 | 38.96 | 21.98 | 237.09 | 2.31 | 8.33 | 8.03E-51 |
| Distractibility | Psychiatric disorders | 20 | 11.41 | 6.84 | 19.02 | 11.38 | 139.61 | 1.94 | 5.64 | 1.53E-29 |
| Frustration tolerance decreased | Psychiatric disorders | 19 | 4.24 | 2.63 | 6.85 | 4.23 | 41.46 | 1.06 | 2.58 | 5.68E-08 |
| Oromandibular dystonia | Nervous system disorders | 18 | 9.58 | 5.65 | 16.23 | 9.56 | 106.18 | 1.74 | 4.88 | 3.19E-22 |
| Raynaud's phenomenon | Vascular disorders | 18 | 7.87 | 4.70 | 13.19 | 7.86 | 86.47 | 1.59 | 4.22 | 6.73E-18 |
| Application site dryness | General disorders and administration site conditions | 18 | 33.82 | 17.42 | 65.64 | 33.75 | 277.85 | 2.36 | 9.71 | 1.04E-59 |
| Acute myocardial infarction | Cardiac disorders | 18 | 20.53 | 11.35 | 37.13 | 20.49 | 203.15 | 2.18 | 7.83 | 2.03E-43 |
| Application site vesicles | General disorders and administration site conditions | 18 | 18.54 | 10.37 | 33.16 | 18.51 | 188.62 | 2.14 | 7.43 | 3.01E-40 |
| Weight gain poor | Metabolism and nutrition disorders | 17 | 4.45 | 2.68 | 7.39 | 4.44 | 39.80 | 1.05 | 2.63 | 1.33E-07 |
| Onychophagia | Psychiatric disorders | 17 | 17.51 | 9.69 | 31.65 | 17.48 | 170.60 | 2.06 | 7.09 | 2.59E-36 |
| Application site erosion | General disorders and administration site conditions | 17 | 108.58 | 40.05 | 294.37 | 108.37 | 411.03 | 2.50 | 11.03 | 1.04E-88 |
| Coeliac disease | Gastrointestinal disorders | 16 | 3.65 | 2.17 | 6.12 | 3.64 | 27.54 | 0.82 | 2.19 | 7.27E-05 |
| Application site burn | General disorders and administration site conditions | 16 | 10.87 | 6.16 | 19.17 | 10.85 | 106.76 | 1.74 | 5.19 | 2.37E-22 |
| Dermatillomania | Psychiatric disorders | 15 | 9.58 | 5.38 | 17.06 | 9.56 | 88.48 | 1.61 | 4.68 | 2.43E-18 |
| Excessive eye blinking | Eye disorders | 15 | 4.99 | 2.89 | 8.60 | 4.98 | 41.28 | 1.09 | 2.82 | 6.24E-08 |
| Myocardial infarction | Cardiac disorders | 15 | 3.86 | 2.26 | 6.60 | 3.86 | 28.31 | 0.84 | 2.26 | 4.88E-05 |
| Application site reaction | General disorders and administration site conditions | 15 | 11.14 | 6.18 | 20.05 | 11.12 | 102.42 | 1.70 | 5.20 | 2.12E-21 |
| Bruxism | Psychiatric disorders | 14 | 4.25 | 2.44 | 7.43 | 4.25 | 30.71 | 0.90 | 2.42 | 1.42E-05 |
| Anal incontinence | Gastrointestinal disorders | 14 | 3.55 | 2.04 | 6.16 | 3.54 | 22.99 | 0.72 | 2.07 | 0.00077 |
| Impulse-control disorder | Psychiatric disorders | 13 | 4.88 | 2.72 | 8.75 | 4.87 | 34.74 | 0.98 | 2.67 | 1.78E-06 |
| Erection increased | Reproductive system and breast disorders | 11 | 5.75 | 3.03 | 10.94 | 5.75 | 36.55 | 1.00 | 2.93 | 7.02E-07 |
| Obsessive thoughts | Psychiatric disorders | 11 | 3.99 | 2.13 | 7.47 | 3.98 | 21.86 | 0.69 | 2.16 | 0.001386 |
| Illusion | Psychiatric disorders | 10 | 9.97 | 4.90 | 20.29 | 9.96 | 61.42 | 1.28 | 4.32 | 2.18E-12 |
| Cataract subcapsular | Eye disorders | 10 | 7.98 | 3.99 | 15.95 | 7.97 | 48.76 | 1.15 | 3.68 | 1.37E-09 |
| Application site haemorrhage | General disorders and administration site conditions | 10 | 53.18 | 19.32 | 146.36 | 53.12 | 191.78 | 1.74 | 8.81 | 6.14E-41 |
| Underweight | Metabolism and nutrition disorders | 10 | 5.91 | 3.01 | 11.60 | 5.90 | 34.36 | 0.94 | 2.92 | 2.16E-06 |
| Delayed puberty | Endocrine disorders | 10 | 4.91 | 2.52 | 9.55 | 4.90 | 26.94 | 0.80 | 2.51 | 9.94E-05 |
| Appetite disorder | Metabolism and nutrition disorders | 10 | 4.43 | 2.29 | 8.59 | 4.43 | 23.30 | 0.72 | 2.30 | 0.000656 |
| Dysphoria | Psychiatric disorders | 10 | 4.31 | 2.23 | 8.35 | 4.31 | 22.38 | 0.69 | 2.25 | 0.00106 |
| Tension | Psychiatric disorders | 10 | 3.84 | 1.99 | 7.41 | 3.84 | 18.75 | 0.59 | 2.04 | 0.007041 |
| Application site oedema | General disorders and administration site conditions | 9 | 95.72 | 25.91 | 353.62 | 95.62 | 210.67 | 1.63 | 8.26 | 4.64E-45 |
| Vitiligo | Skin and subcutaneous tissue disorders | 9 | 7.56 | 3.65 | 15.63 | 7.55 | 41.35 | 1.02 | 3.43 | 6.03E-08 |
| Abnormal loss of weight | Metabolism and nutrition disorders | 9 | 5.13 | 2.54 | 10.37 | 5.12 | 25.73 | 0.75 | 2.52 | 0.000186 |
| Psychotic symptom | Psychiatric disorders | 9 | 4.71 | 2.34 | 9.48 | 4.70 | 22.87 | 0.69 | 2.35 | 0.000821 |
| Application site exfoliation | General disorders and administration site conditions | 9 | 14.36 | 6.54 | 31.54 | 14.34 | 77.05 | 1.33 | 5.28 | 7.89E-16 |
| Compulsions | Psychiatric disorders | 8 | 9.45 | 4.29 | 20.81 | 9.44 | 46.60 | 1.03 | 3.88 | 4.12E-09 |
| Livedo reticularis | Skin and subcutaneous tissue disorders | 8 | 4.91 | 2.33 | 10.33 | 4.90 | 21.55 | 0.63 | 2.35 | 0.001631 |
| Stereotypy | Psychiatric disorders | 8 | 4.25 | 2.03 | 8.90 | 4.25 | 17.55 | 0.51 | 2.09 | 0.013272 |
| Regressive behaviour | Psychiatric disorders | 7 | 7.44 | 3.27 | 16.95 | 7.44 | 31.63 | 0.77 | 3.12 | 8.84E-06 |
| Phobia | Psychiatric disorders | 7 | 6.20 | 2.76 | 13.94 | 6.20 | 25.55 | 0.66 | 2.72 | 0.000204 |
| Daydreaming | Psychiatric disorders | 7 | 5.58 | 2.50 | 12.46 | 5.58 | 22.39 | 0.60 | 2.50 | 0.001055 |
| Obsessive-compulsive symptom | Psychiatric disorders | 7 | 5.45 | 2.44 | 12.14 | 5.44 | 21.68 | 0.58 | 2.45 | 0.001523 |
| Polydipsia psychogenic | Psychiatric disorders | 7 | 44.66 | 14.17 | 140.73 | 44.62 | 124.38 | 1.22 | 7.34 | 3.29E-26 |
| Application site scab | General disorders and administration site conditions | 7 | 27.91 | 10.12 | 76.99 | 27.89 | 96.79 | 1.18 | 6.56 | 3.65E-20 |
| Morose | Psychiatric disorders | 7 | 22.33 | 8.50 | 58.67 | 22.31 | 83.82 | 1.14 | 6.03 | 2.56E-17 |
| Alice in wonderland syndrome | Psychiatric disorders | 7 | 13.95 | 5.74 | 33.93 | 13.94 | 58.52 | 1.03 | 4.76 | 9.54E-12 |
| Wolff-Parkinson-White syndrome | Cardiac disorders | 6 | 9.57 | 3.84 | 23.83 | 9.56 | 35.39 | 0.72 | 3.53 | 1.28E-06 |
| Gilbert's syndrome | Congenital, familial and genetic disorders | 6 | 8.32 | 3.39 | 20.44 | 8.31 | 30.62 | 0.66 | 3.21 | 1.48E-05 |
| Heat stroke | Injury, poisoning and procedural complications | 6 | 6.38 | 2.65 | 15.33 | 6.37 | 22.66 | 0.53 | 2.63 | 0.000915 |
| Tongue biting | Nervous system disorders | 6 | 4.56 | 1.94 | 10.72 | 4.55 | 14.56 | 0.32 | 2.01 | 0.064228 |
| Benign rolandic epilepsy | Nervous system disorders | 6 | 27.34 | 9.19 | 81.36 | 27.32 | 81.92 | 0.96 | 6.09 | 6.7E-17 |
| Application site warmth | General disorders and administration site conditions | 6 | 23.92 | 8.30 | 68.96 | 23.90 | 75.25 | 0.94 | 5.81 | 1.96E-15 |
| Change in sustained attention | Psychiatric disorders | 6 | 21.26 | 7.57 | 59.75 | 21.25 | 69.47 | 0.93 | 5.54 | 3.68E-14 |
| Dermal absorption impaired | Skin and subcutaneous tissue disorders | 6 | 191.37 | 23.04 | 1589.76 | 191.23 | 162.21 | 1.00 | 4.79 | 1.76E-34 |
| Painful erection | Reproductive system and breast disorders | 6 | 17.40 | 6.43 | 47.05 | 17.38 | 59.96 | 0.89 | 5.05 | 4.59E-12 |
| Social anxiety disorder | Psychiatric disorders | 5 | 7.97 | 2.99 | 21.25 | 7.97 | 24.38 | 0.44 | 2.90 | 0.000375 |
| Peripheral vascular disorder | Vascular disorders | 5 | 6.64 | 2.53 | 17.42 | 6.64 | 19.83 | 0.36 | 2.53 | 0.004013 |
| Accommodation disorder | Eye disorders | 5 | 5.31 | 2.06 | 13.70 | 5.31 | 15.00 | 0.25 | 2.13 | 0.050769 |
| Application site inflammation | General disorders and administration site conditions | 5 | 31.89 | 9.23 | 110.18 | 31.87 | 74.76 | 0.70 | 5.82 | 2.51E-15 |
| Application site dermatitis | General disorders and administration site conditions | 5 | 22.78 | 7.23 | 71.79 | 22.77 | 60.70 | 0.68 | 5.24 | 3.15E-12 |
| Mitral valve disease | Cardiac disorders | 5 | 22.78 | 7.23 | 71.79 | 22.77 | 60.70 | 0.68 | 5.24 | 3.15E-12 |
| Tongue spasm | Gastrointestinal disorders | 5 | 11.39 | 4.10 | 31.63 | 11.38 | 34.89 | 0.56 | 3.68 | 1.65E-06 |
| Water intoxication | Metabolism and nutrition disorders | 4 | 9.81 | 3.20 | 30.10 | 9.81 | 24.19 | 0.23 | 3.03 | 0.000412 |
| Intraventricular haemorrhage | Nervous system disorders | 4 | 9.11 | 3.00 | 27.68 | 9.11 | 22.45 | 0.21 | 2.88 | 0.001019 |
| Application site papules | General disorders and administration site conditions | 4 | 63.77 | 11.68 | 348.24 | 63.74 | 82.35 | 0.35 | 5.30 | 5.4E-17 |
| Dyslalia | Nervous system disorders | 4 | 6.71 | 2.28 | 19.74 | 6.71 | 16.06 | 0.11 | 2.32 | 0.029072 |
| Nail picking | Psychiatric disorders | 4 | 42.52 | 9.51 | 190.00 | 42.50 | 69.46 | 0.36 | 5.37 | 3.68E-14 |
| Vasoconstriction | Vascular disorders | 4 | 18.22 | 5.33 | 62.26 | 18.21 | 41.41 | 0.34 | 4.28 | 5.84E-08 |
| Selective mutism | Psychiatric disorders | 4 | 15.94 | 4.80 | 52.96 | 15.94 | 37.33 | 0.32 | 4.01 | 4.71E-07 |
| Huntington's disease | Congenital, familial and genetic disorders | 4 | 127.55 | 14.25 | 1141.32 | 127.49 | 100.40 | 0.33 | 4.20 | 5.89E-21 |
| Disruptive mood dysregulation disorder | Psychiatric disorders | 4 | 11.59 | 3.69 | 36.42 | 11.59 | 28.38 | 0.27 | 3.36 | 4.7E-05 |
| Vasospasm | Vascular disorders | 4 | 10.63 | 3.43 | 32.96 | 10.62 | 26.16 | 0.25 | 3.19 | 0.000149 |

**Supplementary Table S6. Case details of 24 methylphenidate-associated coronary artery dissection reports in pediatric patients (aged 6**–**17 years).**

| ISR | Gender | Age in report | Reporter country | Outcome | Indication | Drug name | Role code | Route | Dosis | Event |
| --- | --- | --- | --- | --- | --- | --- | --- | --- | --- | --- |
| 189892661 | M | 6 | DE | Hospitalization - Initial or Prolonged/ Other | attention deficit hyperactivity disorder | methylphenidate | Primary Suspect | Oral | 5 mg/day | acute myocardial infarction / angina pectoris / coronary artery dissection |
|  |  |  |  |  | – |  | Secondary Suspect |  | 10 mg/day |  |
| 189253821 | M | 6 | DE | Hospitalization - Initial or Prolonged/ Other | attention deficit hyperactivity disorder | methylphenidate | Primary Suspect | Oral | 5 mg/day | acute coronary syndrome / angina pectoris / coronary artery dissection / coronary artery occlusion / myocardial infarction |
|  |  |  |  |  | – |  | Secondary Suspect |  | 10 mg/day |  |
| 190238341 | M | 6 | DE | Hospitalization - Initial or Prolonged | attention deficit hyperactivity disorder | methylphenidate | Primary Suspect | Oral | 5 mg/day | acute myocardial infarction / coronary artery dissection / mitral valve incompetence |
|  |  |  |  |  | – |  | Secondary Suspect |  | 10 mg/day |  |
| 190827681 | M | 6 | DE | Other | attention deficit hyperactivity disorder | methylphenidate | Primary Suspect | – | – | acute myocardial infarction / coronary artery dissection |
| 236029861 | M | 6 | DK | Other | attention deficit hyperactivity disorder | methylphenidate | Primary Suspect | – | – | acute myocardial infarction / coronary artery dissection |
| 241311191 | M | 6 | FR | Life-Threatening/ Other | attention deficit hyperactivity disorder | methylphenidate | Primary Suspect | – | 10 mg/day | coronary artery dissection / dyskinesia / mitral valve incompetence / myocardial infarction / thrombosis |
| 189423151 | M | 6 | DE | Life-Threatening/ Hospitalization - Initial or Prolonged | attention deficit hyperactivity disorder | methylphenidate | Primary Suspect | Oral | 5 mg/day | coronary artery dissection / myocardial infarction |
|  |  |  |  |  | – |  | Secondary Suspect |  | 10 mg/day |  |
| 211801511 | M | 6 | DE | Hospitalization - Initial or Prolonged/ Other | attention deficit hyperactivity disorder | methylphenidate | Primary Suspect | Oral | 10 mg/day | coronary artery dissection / myocardial infarction |
|  |  |  |  |  | – |  | Secondary Suspect |  | 5 mg/day |  |
| 236786741 | M | 6 | DE | Life-Threatening/ Hospitalization - Initial or Prolonged/ Other | attention deficit hyperactivity disorder | methylphenidate | Primary Suspect | Oral | – | acute myocardial infarction / coronary artery dissection |
|  |  |  |  |  | – |  | Secondary Suspect |  | 5 mg/day |  |
| 189632871 | M | 6 | DE | Hospitalization - Initial or Prolonged | attention deficit hyperactivity disorder | methylphenidate | Primary Suspect | Oral | 5 mg/day | acute myocardial infarction / coronary artery dissection / mitral valve incompetence |
|  |  |  |  |  | – |  | Secondary Suspect |  | 10 mg/day |  |
| 211801631 | M | 6 | DE | Other | attention deficit hyperactivity disorder | methylphenidate | Primary Suspect | – | – | coronary artery dissection / myocardial infarction |
| 189534321 | M | 6 | DE | Other | attention deficit hyperactivity disorder | methylphenidate | Primary Suspect | – | – | coronary artery dissection / myocardial infarction |
| 189861941 | M | 6 | DE | Hospitalization - Initial or Prolonged/ Other | attention deficit hyperactivity disorder | methylphenidate | Primary Suspect | Oral | 5 mg/day | acute myocardial infarction / coronary artery dissection / mitral valve incompetence |
|  |  |  |  |  | – |  | Secondary Suspect |  | 10 mg/day |  |
| 189372081 | M | 6 | US | Life-Threatening/ Hospitalization - Initial or Prolonged/ Other | attention deficit hyperactivity disorder | methylphenidate | Primary Suspect | Oral | – | coronary artery dissection / dyskinesia / mitral valve incompetence / myocardial infarction / thrombosis |
| 211413171 | M | 6 | DE | Other | attention deficit hyperactivity disorder | methylphenidate | Primary Suspect | Oral | 5 mg/day | acute myocardial infarction / coronary artery dissection |
|  |  |  |  |  | – |  | Secondary Suspect |  | 10 mg/day |  |
| 222472261 | M | 6 | DE | Other | attention deficit hyperactivity disorder | methylphenidate | Primary Suspect | – | – | coronary artery dissection / myocardial infarction |
| 189588461 | M | 6 | DE | Life-Threatening/ Hospitalization - Initial or Prolonged | attention deficit hyperactivity disorder | methylphenidate | Primary Suspect | Oral | 5 mg/day | coronary artery dissection / myocardial infarction |
|  |  |  |  |  | – |  | Secondary Suspect |  | 10 mg/day |  |
| 190341432 | M | 6 | DE | Life-Threatening/ Hospitalization - Initial or Prolonged/ Other | attention deficit hyperactivity disorder | methylphenidate | Primary Suspect | Oral | 0.14 mg/kg | acute myocardial infarction / coronary artery dissection |
|  |  |  |  |  | – |  | Secondary Suspect | – | – |  |
| 190102482 | M | 6 | DE | Hospitalization - Initial or Prolonged/ Other | attention deficit hyperactivity disorder | methylphenidate | Primary Suspect | Oral | – | acute myocardial infarction / coronary artery dissection |
| 190216901 | M | 6 | DE | Hospitalization - Initial or Prolonged/ Other | attention deficit hyperactivity disorder | methylphenidate | Primary Suspect | Oral | – | acute myocardial infarction / coronary artery dissection |
|  |  |  |  |  | – |  | Secondary Suspect |  | – |  |
|  |  |  |  |  | acute myocardial infarction | eptifibatide | Interacting | – | – |  |
|  |  |  |  |  |  | aspirin |  | – | – |  |
|  |  |  |  |  |  | bisoprolol |  | Oral | – |  |
|  |  |  |  |  |  | lisinopril |  | – | – |  |
|  |  |  |  |  |  | prasugrel |  | – | – |  |
| 239385671 | M | 6 | DE | Hospitalization - Initial or Prolonged/ Other | attention deficit hyperactivity disorder | methylphenidate | Primary Suspect | Oral | 5 mg/day | acute coronary syndrome / acute myocardial infarction / coronary artery dissection |
|  |  |  |  |  | – |  | Secondary Suspect |  | 10 mg/day |  |
| 236468252 | M | 6 | DE | Life-Threatening/ Hospitalization - Initial or Prolonged/ Other | attention deficit hyperactivity disorder | methylphenidate | Primary Suspect | Oral | – | acute myocardial infarction / coronary artery dissection |
|  |  |  |  |  | – |  | Secondary Suspect |  | 5 mg/day |  |
| 189611354 | M | 6 | - | Life-Threatening/ Hospitalization - Initial or Prolonged | attention deficit hyperactivity disorder | methylphenidate (concerta) | Primary Suspect | Oral | – | acute myocardial infarction / coronary artery dissection |
|  |  |  |  |  |  | methylphenidate | Secondary Suspect |  |  |  |
| 189707642 | M | 6 | DE | Life-Threatening/ Hospitalization - Initial or Prolonged/ Other | attention deficit hyperactivity disorder | methylphenidate | Primary Suspect | Oral | 10 mg/day | acute myocardial infarction / coronary artery dissection |
|  |  |  |  |  | – |  | Secondary Suspect |  | 5 mg/day |  |

**Supplementary Table S7. Positive ADR signals associated with methylphenidate in male pediatric patients.**

| PT | SOC | Case reports | ROR | 95%CI | | PRR | χ^2^ | IC025 | EBGM05 | *P*_adj (Bonferroni) |
| --- | --- | --- | --- | --- | --- | --- | --- | --- | --- | --- |
|  |  |  |  | lower | upper |  |  |  |  |  |
| Abnormal behaviour | Psychiatric disorders | 308 | 3.08 | 2.73 | 3.48 | 2.98 | 360.71 | 1.26 | 2.47 | 9.33E-78 |
| Aggression | Psychiatric disorders | 304 | 2.56 | 2.27 | 2.90 | 2.49 | 247.26 | 1.04 | 2.11 | 4.86E-53 |
| Application site erythema | General disorders and administration site conditions | 284 | 142.63 | 103.71 | 196.14 | 136.15 | 5123.29 | 3.93 | 14.65 | 0 |
| Decreased appetite | Metabolism and nutrition disorders | 267 | 3.36 | 2.94 | 3.83 | 3.26 | 366.78 | 1.36 | 2.65 | 4.44E-79 |
| Disturbance in attention | Nervous system disorders | 224 | 5.71 | 4.92 | 6.63 | 5.54 | 665.15 | 1.96 | 4.06 | 5.4E-144 |
| Insomnia | Psychiatric disorders | 218 | 2.92 | 2.53 | 3.38 | 2.86 | 234.73 | 1.18 | 2.34 | 2.62E-50 |
| Agitation | Psychiatric disorders | 205 | 2.75 | 2.37 | 3.18 | 2.69 | 195.25 | 1.09 | 2.21 | 1.07E-41 |
| Tic | Psychiatric disorders | 152 | 4.09 | 3.43 | 4.87 | 4.01 | 290.89 | 1.54 | 3.05 | 1.51E-62 |
| Psychomotor hyperactivity | Nervous system disorders | 145 | 4.10 | 3.42 | 4.90 | 4.02 | 278.61 | 1.54 | 3.05 | 7.15E-60 |
| Application site pruritus | General disorders and administration site conditions | 143 | 96.46 | 65.69 | 141.64 | 94.26 | 2415.87 | 3.68 | 13.09 | 0 |
| Application site irritation | General disorders and administration site conditions | 99 | 96.44 | 60.72 | 153.18 | 94.92 | 1674.24 | 3.55 | 12.27 | 0 |
| Dyskinesia | Nervous system disorders | 99 | 3.10 | 2.50 | 3.83 | 3.06 | 120.73 | 1.15 | 2.34 | 2.07E-25 |
| Disturbance in social behaviour | Psychiatric disorders | 85 | 39.50 | 27.57 | 56.60 | 38.98 | 1105.49 | 3.22 | 10.61 | 1E-239 |
| Application site pain | General disorders and administration site conditions | 81 | 13.20 | 10.00 | 17.44 | 13.04 | 557.40 | 2.57 | 6.69 | 1.5E-120 |
| Growth retardation | Musculoskeletal and connective tissue disorders | 75 | 3.79 | 2.96 | 4.85 | 3.76 | 129.31 | 1.34 | 2.72 | 2.75E-27 |
| Hallucination, visual | Psychiatric disorders | 74 | 4.66 | 3.62 | 6.00 | 4.62 | 172.61 | 1.57 | 3.21 | 9.43E-37 |
| Restlessness | Psychiatric disorders | 74 | 3.23 | 2.52 | 4.12 | 3.20 | 97.51 | 1.15 | 2.37 | 2.54E-20 |
| Psychotic disorder | Psychiatric disorders | 67 | 2.92 | 2.26 | 3.78 | 2.90 | 73.72 | 1.01 | 2.16 | 4.26E-15 |
| Application site rash | General disorders and administration site conditions | 55 | 55.73 | 33.69 | 92.20 | 55.25 | 809.91 | 3.13 | 10.49 | 1.8E-175 |
| Impulsive behaviour | Psychiatric disorders | 49 | 4.30 | 3.16 | 5.85 | 4.27 | 102.30 | 1.37 | 2.87 | 2.26E-21 |
| Withdrawal syndrome | General disorders and administration site conditions | 48 | 4.03 | 2.95 | 5.49 | 4.00 | 91.05 | 1.29 | 2.72 | 6.62E-19 |
| Hallucination, auditory | Psychiatric disorders | 48 | 2.96 | 2.18 | 4.01 | 2.94 | 54.20 | 0.94 | 2.10 | 8.57E-11 |
| Logorrhoea | Psychiatric disorders | 40 | 8.00 | 5.56 | 11.53 | 7.96 | 176.92 | 1.91 | 4.46 | 1.08E-37 |
| Arrhythmia | Cardiac disorders | 38 | 3.49 | 2.47 | 4.92 | 3.47 | 57.48 | 1.07 | 2.34 | 1.61E-11 |
| Sudden death | General disorders and administration site conditions | 34 | 9.24 | 6.17 | 13.83 | 9.19 | 173.08 | 1.96 | 4.78 | 7.45E-37 |
| Priapism | Reproductive system and breast disorders | 31 | 4.18 | 2.84 | 6.15 | 4.16 | 62.35 | 1.19 | 2.64 | 1.36E-12 |
| Coronary artery dissection | Cardiac disorders | 24 | 508.21 | 68.74 | 3757.34 | 506.25 | 484.17 | 2.71 | 3.98 | 1.3E-104 |
| Choreoathetosis | Nervous system disorders | 23 | 14.75 | 8.66 | 25.14 | 14.70 | 173.14 | 2.04 | 5.81 | 7.2E-37 |
| Application site swelling | General disorders and administration site conditions | 22 | 33.26 | 17.01 | 65.04 | 33.15 | 266.81 | 2.34 | 7.70 | 2.66E-57 |
| Hyperkinesia | Nervous system disorders | 22 | 21.16 | 11.71 | 38.24 | 21.09 | 210.62 | 2.18 | 6.73 | 4.76E-45 |
| Tourette's disorder | Congenital, familial and genetic disorders | 22 | 5.11 | 3.21 | 8.15 | 5.10 | 58.44 | 1.24 | 2.91 | 9.93E-12 |
| Formication | Nervous system disorders | 20 | 13.65 | 7.78 | 23.96 | 13.61 | 142.07 | 1.90 | 5.41 | 4.44E-30 |
| Trismus | Musculoskeletal and connective tissue disorders | 19 | 5.15 | 3.12 | 8.51 | 5.14 | 50.96 | 1.17 | 2.84 | 4.47E-10 |
| Acute myocardial infarction | Cardiac disorders | 18 | 22.40 | 11.54 | 43.48 | 22.33 | 178.22 | 2.03 | 6.52 | 5.62E-38 |
| Oromandibular dystonia | Nervous system disorders | 18 | 10.57 | 6.00 | 18.63 | 10.55 | 103.74 | 1.68 | 4.58 | 1.09E-21 |
| Euphoric mood | Psychiatric disorders | 18 | 4.70 | 2.82 | 7.83 | 4.69 | 42.75 | 1.06 | 2.62 | 2.94E-08 |
| Dysphemia | Psychiatric disorders | 18 | 4.64 | 2.78 | 7.73 | 4.63 | 42.04 | 1.05 | 2.59 | 4.23E-08 |
| Hallucination, tactile | Psychiatric disorders | 17 | 13.31 | 7.25 | 24.44 | 13.28 | 118.51 | 1.76 | 5.14 | 6.35E-25 |
| Pulmonary hypertension | Respiratory, thoracic and mediastinal disorders | 17 | 4.04 | 2.40 | 6.79 | 4.03 | 32.53 | 0.89 | 2.29 | 5.55E-06 |
| Application site urticaria | General disorders and administration site conditions | 16 | 169.18 | 38.89 | 735.97 | 168.75 | 296.49 | 2.23 | 5.74 | 9.06E-64 |
| Cardiac valve disease | Cardiac disorders | 16 | 18.80 | 9.58 | 36.88 | 18.75 | 142.37 | 1.86 | 5.92 | 3.82E-30 |
| Trichotillomania | Psychiatric disorders | 15 | 11.74 | 6.24 | 22.09 | 11.72 | 94.57 | 1.60 | 4.65 | 1.12E-19 |
| Mitral valve incompetence | Cardiac disorders | 15 | 6.75 | 3.77 | 12.07 | 6.73 | 55.52 | 1.25 | 3.29 | 4.37E-11 |
| Myocardial infarction | Cardiac disorders | 15 | 5.37 | 3.05 | 9.48 | 5.36 | 42.47 | 1.07 | 2.79 | 3.4E-08 |
| Visual acuity reduced | Eye disorders | 15 | 3.73 | 2.15 | 6.46 | 3.72 | 25.40 | 0.74 | 2.09 | 0.00022 |
| Application site vesicles | General disorders and administration site conditions | 14 | 24.66 | 11.40 | 53.34 | 24.61 | 146.38 | 1.83 | 6.24 | 5.07E-31 |
| Raynaud's phenomenon | Vascular disorders | 14 | 10.57 | 5.56 | 20.08 | 10.55 | 80.68 | 1.48 | 4.30 | 1.26E-16 |
| Excessive eye blinking | Eye disorders | 14 | 4.17 | 2.35 | 7.39 | 4.16 | 28.08 | 0.81 | 2.25 | 5.51E-05 |
| Application site dryness | General disorders and administration site conditions | 13 | 68.70 | 22.39 | 210.75 | 68.55 | 203.65 | 1.92 | 6.61 | 1.58E-43 |
| Onychophagia | Psychiatric disorders | 13 | 13.74 | 6.83 | 27.63 | 13.71 | 92.86 | 1.55 | 4.85 | 2.65E-19 |
| Coeliac disease | Gastrointestinal disorders | 13 | 5.28 | 2.88 | 9.71 | 5.27 | 36.03 | 0.96 | 2.66 | 9.18E-07 |
| Application site burn | General disorders and administration site conditions | 12 | 19.51 | 8.90 | 42.77 | 19.47 | 109.36 | 1.60 | 5.50 | 6.4E-23 |
| Distractibility | Psychiatric disorders | 11 | 5.67 | 2.91 | 11.03 | 5.66 | 33.28 | 0.90 | 2.68 | 3.77E-06 |
| Precocious puberty | Endocrine disorders | 11 | 4.47 | 2.33 | 8.57 | 4.46 | 24.40 | 0.71 | 2.24 | 0.00037 |
| Application site reaction | General disorders and administration site conditions | 10 | 35.21 | 12.79 | 96.91 | 35.16 | 124.46 | 1.52 | 5.92 | 3.17E-26 |
| Dermatillomania | Psychiatric disorders | 10 | 6.21 | 3.07 | 12.58 | 6.20 | 33.74 | 0.89 | 2.78 | 2.98E-06 |
| Impulse-control disorder | Psychiatric disorders | 10 | 4.49 | 2.27 | 8.90 | 4.49 | 22.36 | 0.65 | 2.19 | 0.001068 |
| Application site discolouration | General disorders and administration site conditions | 9 | 21.12 | 8.38 | 53.23 | 21.09 | 86.15 | 1.30 | 5.10 | 7.91E-18 |
| Illusion | Psychiatric disorders | 8 | 10.56 | 4.52 | 24.68 | 10.55 | 46.10 | 0.96 | 3.62 | 5.32E-09 |
| Vitiligo | Skin and subcutaneous tissue disorders | 8 | 8.04 | 3.56 | 18.17 | 8.04 | 35.69 | 0.84 | 3.08 | 1.09E-06 |
| Gastrointestinal pain | Gastrointestinal disorders | 8 | 4.97 | 2.30 | 10.74 | 4.96 | 20.50 | 0.55 | 2.21 | 0.002819 |
| Application site haemorrhage | General disorders and administration site conditions | 7 | 147.82 | 18.18 | 1201.69 | 147.65 | 127.46 | 1.10 | 3.35 | 6.96E-27 |
| Application site scab | General disorders and administration site conditions | 7 | 36.95 | 10.81 | 126.27 | 36.91 | 88.95 | 1.06 | 5.03 | 1.92E-18 |
| Psychotic symptom | Psychiatric disorders | 7 | 7.04 | 2.99 | 16.56 | 7.03 | 27.17 | 0.64 | 2.70 | 8.83E-05 |
| Abnormal loss of weight | Metabolism and nutrition disorders | 7 | 6.72 | 2.87 | 15.73 | 6.71 | 25.82 | 0.61 | 2.62 | 0.000178 |
| Obsessive-compulsive symptom | Psychiatric disorders | 7 | 5.10 | 2.23 | 11.64 | 5.09 | 18.55 | 0.45 | 2.15 | 0.007848 |
| Acute psychosis | Psychiatric disorders | 7 | 4.77 | 2.10 | 10.83 | 4.76 | 16.98 | 0.41 | 2.05 | 0.017848 |
| Application site oedema | General disorders and administration site conditions | 6 | 126.68 | 15.25 | 1052.46 | 126.56 | 106.78 | 0.87 | 3.22 | 2.35E-22 |
| Alice in wonderland syndrome | Psychiatric disorders | 6 | 63.34 | 12.78 | 313.90 | 63.28 | 91.95 | 0.86 | 4.34 | 4.2E-19 |
| Benign rolandic epilepsy | Nervous system disorders | 6 | 42.23 | 10.56 | 168.89 | 42.19 | 80.43 | 0.85 | 4.62 | 1.43E-16 |
| Painful erection | Reproductive system and breast disorders | 6 | 11.52 | 4.26 | 31.15 | 11.51 | 37.25 | 0.66 | 3.39 | 4.93E-07 |
| Gilbert's syndrome | Congenital, familial and genetic disorders | 6 | 7.45 | 2.94 | 18.90 | 7.44 | 24.75 | 0.51 | 2.64 | 0.000309 |
| Daydreaming | Psychiatric disorders | 6 | 6.03 | 2.43 | 14.95 | 6.03 | 19.57 | 0.41 | 2.30 | 0.004588 |
| Dermal absorption impaired | Skin and subcutaneous tissue disorders | 5 | 105.55 | 12.33 | 903.64 | 105.47 | 86.23 | 0.58 | 3.05 | 7.56E-18 |
| Mitral valve disease | Cardiac disorders | 5 | 26.39 | 7.08 | 98.29 | 26.37 | 54.24 | 0.56 | 4.08 | 8.41E-11 |
| Tongue spasm | Gastrointestinal disorders | 5 | 26.39 | 7.08 | 98.29 | 26.37 | 54.24 | 0.56 | 4.08 | 8.41E-11 |
| Social anxiety disorder | Psychiatric disorders | 5 | 17.59 | 5.37 | 57.66 | 17.58 | 42.64 | 0.52 | 3.72 | 3.11E-08 |
| Application site exfoliation | General disorders and administration site conditions | 5 | 13.19 | 4.31 | 40.34 | 13.18 | 34.65 | 0.47 | 3.34 | 1.87E-06 |
| Wolff-Parkinson-White syndrome | Cardiac disorders | 5 | 6.21 | 2.29 | 16.83 | 6.20 | 16.87 | 0.23 | 2.18 | 0.018952 |
| Peripheral vascular disorder | Vascular disorders | 5 | 5.86 | 2.18 | 15.80 | 5.86 | 15.77 | 0.21 | 2.10 | 0.033794 |
| Vasospasm | Vascular disorders | 4 | 42.21 | 7.73 | 230.52 | 42.19 | 53.62 | 0.23 | 3.56 | 1.15E-10 |
| Disruptive mood dysregulation disorder | Psychiatric disorders | 4 | 21.11 | 5.28 | 84.41 | 21.09 | 38.28 | 0.22 | 3.46 | 2.89E-07 |
| Selective mutism | Psychiatric disorders | 4 | 21.11 | 5.28 | 84.41 | 21.09 | 38.28 | 0.22 | 3.46 | 2.89E-07 |
| Water intoxication | Metabolism and nutrition disorders | 4 | 21.11 | 5.28 | 84.41 | 21.09 | 38.28 | 0.22 | 3.46 | 2.89E-07 |
| Mitral valve prolapse | Cardiac disorders | 4 | 12.06 | 3.53 | 41.21 | 12.05 | 25.80 | 0.16 | 2.87 | 0.000179 |
| Accommodation disorder | Eye disorders | 4 | 6.49 | 2.12 | 19.92 | 6.49 | 14.21 | 0.003 | 2.03 | 0.077411 |

**Supplementary Table S8. Positive ADR signals associated with methylphenidate in female pediatric patients.**

| PT | SOC | Case reports | ROR | 95%CI | | PRR | χ^2^ | IC025 | EBGM05 | *P*_adj (Bonferroni) |
| --- | --- | --- | --- | --- | --- | --- | --- | --- | --- | --- |
|  |  |  |  | lower | upper |  |  |  |  |  |
| Application site erythema | General disorders and administration site conditions | 134 | 106.80 | 80.77 | 141.21 | 100.31 | 4971.46 | 4.58 | 30.41 | 0 |
| Decreased appetite | Metabolism and nutrition disorders | 101 | 5.10 | 4.15 | 6.28 | 4.91 | 293.96 | 1.85 | 3.88 | 3.23E-63 |
| Agitation | Psychiatric disorders | 94 | 5.37 | 4.33 | 6.66 | 5.18 | 294.85 | 1.91 | 4.05 | 2.07E-63 |
| Abnormal behaviour | Psychiatric disorders | 89 | 4.88 | 3.92 | 6.09 | 4.73 | 244.74 | 1.78 | 3.71 | 1.72E-52 |
| Insomnia | Psychiatric disorders | 84 | 4.48 | 3.58 | 5.62 | 4.35 | 204.07 | 1.66 | 3.42 | 1.28E-43 |
| Disturbance in attention | Nervous system disorders | 79 | 9.67 | 7.60 | 12.30 | 9.36 | 512.99 | 2.57 | 6.74 | 6.7E-111 |
| Aggression | Psychiatric disorders | 76 | 3.99 | 3.15 | 5.05 | 3.88 | 154.29 | 1.50 | 3.04 | 9.47E-33 |
| Tachycardia | Cardiac disorders | 73 | 3.07 | 2.42 | 3.90 | 3.00 | 94.00 | 1.15 | 2.38 | 1.49E-19 |
| Application site pruritus | General disorders and administration site conditions | 68 | 74.63 | 52.35 | 106.39 | 72.34 | 2183.44 | 4.07 | 24.92 | 0 |
| Psychomotor hyperactivity | Nervous system disorders | 51 | 8.37 | 6.23 | 11.25 | 8.20 | 285.02 | 2.28 | 5.74 | 2.86E-61 |
| Hypertension | Vascular disorders | 50 | 3.68 | 2.76 | 4.92 | 3.62 | 90.16 | 1.31 | 2.73 | 1.04E-18 |
| Irritability | Psychiatric disorders | 49 | 4.26 | 3.18 | 5.71 | 4.18 | 111.72 | 1.48 | 3.11 | 1.95E-23 |
| Application site irritation | General disorders and administration site conditions | 47 | 80.88 | 52.29 | 125.11 | 79.16 | 1574.22 | 3.82 | 24.23 | 0 |
| Mydriasis | Eye disorders | 41 | 4.08 | 2.97 | 5.61 | 4.02 | 87.76 | 1.38 | 2.94 | 3.5E-18 |
| Application site pain | General disorders and administration site conditions | 38 | 11.48 | 8.10 | 16.27 | 11.29 | 301.12 | 2.48 | 7.23 | 8.91E-65 |
| Hallucination | Psychiatric disorders | 38 | 2.85 | 2.05 | 3.96 | 2.82 | 42.93 | 0.91 | 2.08 | 2.68E-08 |
| Crying | General disorders and administration site conditions | 36 | 3.15 | 2.25 | 4.42 | 3.12 | 49.54 | 1.02 | 2.27 | 9.18E-10 |
| Heart rate increased | Investigations | 36 | 2.91 | 2.08 | 4.08 | 2.88 | 42.42 | 0.92 | 2.11 | 3.48E-08 |
| Depressed mood | Psychiatric disorders | 31 | 3.65 | 2.54 | 5.26 | 3.61 | 55.56 | 1.16 | 2.56 | 4.28E-11 |
| Mood swings | Psychiatric disorders | 31 | 3.12 | 2.17 | 4.48 | 3.09 | 41.81 | 0.96 | 2.20 | 4.77E-08 |
| Tic | Psychiatric disorders | 30 | 6.50 | 4.45 | 9.50 | 6.43 | 124.61 | 1.80 | 4.30 | 2.93E-26 |
| Dyskinesia | Nervous system disorders | 29 | 4.32 | 2.96 | 6.31 | 4.28 | 68.27 | 1.33 | 2.96 | 6.74E-14 |
| Apathy | Psychiatric disorders | 28 | 12.63 | 8.39 | 19.02 | 12.48 | 245.58 | 2.39 | 7.47 | 1.13E-52 |
| Dystonia | Nervous system disorders | 28 | 4.52 | 3.08 | 6.66 | 4.48 | 70.69 | 1.37 | 3.07 | 1.98E-14 |
| Palpitations | Cardiac disorders | 28 | 3.91 | 2.66 | 5.75 | 3.88 | 56.36 | 1.20 | 2.68 | 2.85E-11 |
| Anger | Psychiatric disorders | 27 | 3.05 | 2.06 | 4.49 | 3.02 | 34.91 | 0.89 | 2.11 | 1.63E-06 |
| Disturbance in social behaviour | Psychiatric disorders | 25 | 61.33 | 35.17 | 106.93 | 60.63 | 733.41 | 3.13 | 19.35 | 7.7E-159 |
| Restlessness | Psychiatric disorders | 25 | 5.58 | 3.70 | 8.43 | 5.53 | 85.26 | 1.55 | 3.65 | 1.24E-17 |
| Hallucination, visual | Psychiatric disorders | 25 | 4.64 | 3.08 | 6.98 | 4.59 | 65.50 | 1.35 | 3.08 | 2.74E-13 |
| Body temperature increased | Investigations | 24 | 9.24 | 6.00 | 14.23 | 9.15 | 151.63 | 2.03 | 5.63 | 3.61E-32 |
| Tachypnoea | Respiratory, thoracic and mediastinal disorders | 24 | 5.87 | 3.86 | 8.95 | 5.82 | 87.61 | 1.59 | 3.80 | 3.77E-18 |
| Psychotic disorder | Psychiatric disorders | 24 | 3.67 | 2.42 | 5.55 | 3.64 | 43.45 | 1.07 | 2.47 | 2.06E-08 |
| Hallucination, auditory | Psychiatric disorders | 22 | 3.58 | 2.33 | 5.52 | 3.56 | 38.32 | 1.00 | 2.38 | 2.85E-07 |
| Application site rash | General disorders and administration site conditions | 21 | 44.32 | 25.24 | 77.85 | 43.91 | 510.91 | 2.85 | 16.16 | 1.9E-110 |
| Affect lability | Psychiatric disorders | 19 | 7.35 | 4.56 | 11.85 | 7.29 | 92.19 | 1.68 | 4.44 | 3.72E-19 |
| Precocious puberty | Endocrine disorders | 18 | 14.86 | 8.86 | 24.92 | 14.75 | 185.69 | 2.19 | 7.83 | 1.31E-39 |
| Emotional disorder | Psychiatric disorders | 18 | 3.61 | 2.24 | 5.82 | 3.59 | 31.83 | 0.92 | 2.31 | 7.95E-06 |
| Blood pressure increased | Investigations | 18 | 3.38 | 2.10 | 5.44 | 3.36 | 28.32 | 0.85 | 2.17 | 4.87E-05 |
| Impulsive behaviour | Psychiatric disorders | 16 | 8.57 | 5.07 | 14.48 | 8.51 | 93.08 | 1.70 | 4.89 | 2.37E-19 |
| Mania | Psychiatric disorders | 16 | 5.11 | 3.06 | 8.52 | 5.08 | 48.44 | 1.23 | 3.10 | 1.61E-09 |
| Application site swelling | General disorders and administration site conditions | 14 | 27.55 | 14.64 | 51.87 | 27.38 | 245.24 | 2.24 | 11.29 | 1.34E-52 |
| Growth retardation | Musculoskeletal and connective tissue disorders | 13 | 4.02 | 2.29 | 7.06 | 4.00 | 27.50 | 0.86 | 2.38 | 7.44E-05 |
| Muscle contracture | Musculoskeletal and connective tissue disorders | 12 | 40.64 | 19.55 | 84.47 | 40.42 | 276.89 | 2.16 | 13.37 | 1.69E-59 |
| Feeling jittery | General disorders and administration site conditions | 12 | 7.62 | 4.17 | 13.90 | 7.58 | 60.97 | 1.38 | 4.14 | 2.74E-12 |
| Application site discolouration | General disorders and administration site conditions | 11 | 37.24 | 17.57 | 78.93 | 37.05 | 239.57 | 2.03 | 12.47 | 2.3E-51 |
| Application site urticaria | General disorders and administration site conditions | 10 | 87.01 | 33.09 | 228.81 | 86.62 | 348.53 | 1.98 | 16.15 | 4.19E-75 |
| Logorrhoea | Psychiatric disorders | 10 | 11.94 | 6.05 | 23.55 | 11.89 | 83.42 | 1.51 | 5.72 | 3.14E-17 |
| Euphoric mood | Psychiatric disorders | 10 | 7.08 | 3.67 | 13.64 | 7.05 | 46.55 | 1.18 | 3.71 | 4.23E-09 |
| Hallucinations, mixed | Psychiatric disorders | 10 | 6.28 | 3.27 | 12.05 | 6.25 | 40.02 | 1.08 | 3.34 | 1.19E-07 |
| Delusion | Psychiatric disorders | 10 | 5.16 | 2.70 | 9.85 | 5.14 | 30.76 | 0.92 | 2.80 | 1.38E-05 |
| Distractibility | Psychiatric disorders | 9 | 36.53 | 15.97 | 83.56 | 36.38 | 193.58 | 1.75 | 11.57 | 2.49E-41 |
| Thinking abnormal | Psychiatric disorders | 9 | 3.83 | 1.95 | 7.52 | 3.82 | 17.62 | 0.58 | 2.07 | 0.012783 |
| Bruxism | Psychiatric disorders | 8 | 9.01 | 4.28 | 18.96 | 8.98 | 49.45 | 1.12 | 4.27 | 9.61E-10 |
| Initial insomnia | Psychiatric disorders | 8 | 4.72 | 2.30 | 9.71 | 4.71 | 21.71 | 0.67 | 2.43 | 0.001502 |
| Social avoidant behaviour | Psychiatric disorders | 8 | 3.95 | 1.93 | 8.10 | 3.94 | 16.52 | 0.52 | 2.07 | 0.022759 |
| Application site erosion | General disorders and administration site conditions | 7 | 212.90 | 44.20 | 1025.46 | 212.22 | 327.04 | 1.41 | 12.86 | 2.01E-70 |
| Sudden death | General disorders and administration site conditions | 7 | 10.64 | 4.76 | 23.78 | 10.61 | 51.88 | 1.07 | 4.68 | 2.79E-10 |
| Mitral valve incompetence | Cardiac disorders | 7 | 7.34 | 3.35 | 16.09 | 7.32 | 34.09 | 0.87 | 3.44 | 2.49E-06 |
| Trismus | Musculoskeletal and connective tissue disorders | 7 | 5.75 | 2.65 | 12.50 | 5.74 | 25.02 | 0.71 | 2.78 | 0.000268 |
| Cardiac valve disease | Cardiac disorders | 6 | 30.40 | 11.40 | 81.07 | 30.32 | 113.42 | 1.14 | 9.04 | 8.27E-24 |
| Trichotillomania | Psychiatric disorders | 6 | 9.12 | 3.86 | 21.53 | 9.10 | 37.61 | 0.82 | 3.92 | 4.1E-07 |
| Application site dryness | General disorders and administration site conditions | 5 | 27.62 | 9.59 | 79.57 | 27.56 | 88.00 | 0.86 | 7.95 | 3.09E-18 |
| Dermatillomania | Psychiatric disorders | 5 | 18.99 | 6.95 | 51.88 | 18.95 | 64.78 | 0.81 | 6.33 | 3.97E-13 |
| Hallucination, tactile | Psychiatric disorders | 5 | 11.68 | 4.48 | 30.46 | 11.66 | 40.88 | 0.70 | 4.46 | 7.65E-08 |
| Frustration tolerance decreased | Psychiatric disorders | 5 | 8.21 | 3.22 | 20.91 | 8.19 | 27.83 | 0.56 | 3.36 | 6.27E-05 |
| Application site reaction | General disorders and administration site conditions | 5 | 8.21 | 3.22 | 20.91 | 8.19 | 27.83 | 0.56 | 3.36 | 6.27E-05 |
| Immune thrombocytopenia | Blood and lymphatic system disorders | 5 | 7.06 | 2.80 | 17.85 | 7.05 | 23.27 | 0.49 | 2.96 | 0.000667 |
| Reversible cerebral vasoconstriction syndrome | Nervous system disorders | 5 | 6.75 | 2.68 | 17.02 | 6.74 | 21.99 | 0.47 | 2.84 | 0.001294 |
| Morose | Psychiatric disorders | 4 | 242.98 | 27.15 | 2174.91 | 242.54 | 192.44 | 0.44 | 7.88 | 4.41E-41 |
| Compulsions | Psychiatric disorders | 4 | 48.59 | 13.04 | 181.09 | 48.51 | 103.40 | 0.52 | 9.11 | 1.29E-21 |
| Dyslalia | Nervous system disorders | 4 | 40.50 | 11.42 | 143.61 | 40.42 | 92.28 | 0.53 | 8.55 | 3.56E-19 |
| Onychophagia | Psychiatric disorders | 4 | 24.30 | 7.61 | 77.53 | 24.25 | 63.71 | 0.52 | 6.67 | 6.83E-13 |
| Application site exfoliation | General disorders and administration site conditions | 4 | 20.25 | 6.52 | 62.83 | 20.21 | 54.79 | 0.50 | 5.97 | 6.34E-11 |
| Hyperkinesia | Nervous system disorders | 4 | 18.69 | 6.09 | 57.36 | 18.66 | 51.12 | 0.49 | 5.67 | 4.11E-10 |
| Choreoathetosis | Nervous system disorders | 4 | 14.29 | 4.80 | 42.51 | 14.27 | 39.95 | 0.45 | 4.72 | 1.23E-07 |
| Application site vesicles | General disorders and administration site conditions | 4 | 12.79 | 4.35 | 37.62 | 12.77 | 35.84 | 0.43 | 4.35 | 1.01E-06 |
| Aortic valve incompetence | Cardiac disorders | 4 | 12.15 | 4.15 | 35.57 | 12.13 | 34.04 | 0.42 | 4.18 | 2.56E-06 |
| Ewing's sarcoma | Neoplasms benign, malignant and unspecified (incl cysts and polyps) | 4 | 9.34 | 3.26 | 26.80 | 9.33 | 25.78 | 0.34 | 3.40 | 0.000181 |
| Livedo reticularis | Skin and subcutaneous tissue disorders | 4 | 8.68 | 3.04 | 24.76 | 8.66 | 23.73 | 0.32 | 3.20 | 0.000525 |
| Dysphoria | Psychiatric disorders | 4 | 7.84 | 2.76 | 22.22 | 7.82 | 21.09 | 0.28 | 2.95 | 0.002071 |
| Energy increased | General disorders and administration site conditions | 4 | 7.84 | 2.76 | 22.22 | 7.82 | 21.09 | 0.28 | 2.95 | 0.002071 |
| Application site burn | General disorders and administration site conditions | 4 | 7.14 | 2.53 | 20.15 | 7.13 | 18.88 | 0.24 | 2.72 | 0.006586 |
| Weight gain poor | Metabolism and nutrition disorders | 4 | 5.65 | 2.03 | 15.75 | 5.64 | 13.98 | 0.13 | 2.22 | 0.087573 |
| Raynaud's phenomenon | Vascular disorders | 4 | 5.40 | 1.94 | 15.02 | 5.39 | 13.14 | 0.11 | 2.14 | 0.136832 |
| Application site dermatitis | General disorders and administration site conditions | 3 | 45.54 | 10.19 | 203.59 | 45.48 | 74.57 | 0.06 | 7.54 | 2.77E-15 |
| Application site warmth | General disorders and administration site conditions | 3 | 45.54 | 10.19 | 203.59 | 45.48 | 74.57 | 0.06 | 7.54 | 2.77E-15 |
| Application site haemorrhage | General disorders and administration site conditions | 3 | 36.43 | 8.70 | 152.53 | 36.38 | 64.52 | 0.07 | 6.97 | 4.52E-13 |
| Hypertrophic cardiomyopathy | Congenital, familial and genetic disorders | 3 | 20.24 | 5.48 | 74.81 | 20.21 | 41.09 | 0.07 | 5.16 | 6.87E-08 |
| Regressive behaviour | Psychiatric disorders | 3 | 14.01 | 3.99 | 49.20 | 13.99 | 29.41 | 0.04 | 4.04 | 2.77E-05 |
| Visual snow syndrome | Eye disorders | 3 | 12.14 | 3.51 | 41.97 | 12.13 | 25.53 | 0.02 | 3.64 | 0.000206 |

**Supplementary Table S9. Positive ADR signals associated with methylphenidate in oral immediate-release formulations.**

| PT | SOC | Case reports | ROR | 95%CI | | PRR | χ^2^ | IC025 | EBGM05 | *P*_adj (Bonferroni) |
| --- | --- | --- | --- | --- | --- | --- | --- | --- | --- | --- |
|  |  |  |  | lower | upper |  |  |  |  |  |
| Aggression | Psychiatric disorders | 37 | 5.43 | 3.88 | 7.60 | 5.09 | 122.49 | 1.70 | 3.82 | 8.54E-26 |
| Abnormal behaviour | Psychiatric disorders | 29 | 4.67 | 3.20 | 6.81 | 4.45 | 78.10 | 1.44 | 3.23 | 4.64E-16 |
| Decreased appetite | Metabolism and nutrition disorders | 25 | 4.46 | 2.98 | 6.69 | 4.29 | 63.32 | 1.34 | 3.04 | 8.31E-13 |
| Agitation | Psychiatric disorders | 23 | 4.50 | 2.95 | 6.85 | 4.33 | 59.17 | 1.31 | 3.03 | 6.82E-12 |
| Weight decreased | Investigations | 20 | 4.52 | 2.88 | 7.08 | 4.37 | 52.13 | 1.26 | 2.99 | 2.46E-10 |
| Insomnia | Psychiatric disorders | 20 | 3.83 | 2.44 | 6.00 | 3.71 | 39.86 | 1.06 | 2.54 | 1.29E-07 |
| Heart rate increased | Investigations | 14 | 5.20 | 3.05 | 8.87 | 5.08 | 45.72 | 1.23 | 3.23 | 6.45E-09 |
| Feeling abnormal | General disorders and administration site conditions | 14 | 4.43 | 2.60 | 7.55 | 4.33 | 35.85 | 1.06 | 2.76 | 1.01E-06 |
| Hallucination | Psychiatric disorders | 14 | 4.16 | 2.44 | 7.09 | 4.07 | 32.44 | 0.99 | 2.59 | 5.81E-06 |
| Hallucination,visual | Psychiatric disorders | 13 | 9.95 | 5.71 | 17.34 | 9.71 | 100.21 | 1.78 | 6.01 | 6.47E-21 |
| Syncope | Nervous system disorders | 12 | 4.34 | 2.44 | 7.71 | 4.26 | 29.86 | 0.94 | 2.62 | 2.2E-05 |
| Dyskinesia | Nervous system disorders | 11 | 5.25 | 2.88 | 9.57 | 5.15 | 36.64 | 1.08 | 3.10 | 6.71E-07 |
| Disturbance in attention | Nervous system disorders | 11 | 3.82 | 2.09 | 6.95 | 3.75 | 22.19 | 0.76 | 2.26 | 0.001165 |
| Sudden death | General disorders and administration site conditions | 10 | 38.17 | 20.01 | 72.81 | 37.41 | 332.95 | 2.19 | 20.50 | 1.03E-71 |
| Fall | Injury, poisoning and procedural complications | 10 | 3.84 | 2.05 | 7.19 | 3.78 | 20.40 | 0.70 | 2.22 | 0.002967 |
| Hallucination,auditory | Psychiatric disorders | 9 | 6.66 | 3.43 | 12.92 | 6.56 | 42.01 | 1.14 | 3.73 | 4.29E-08 |
| Unresponsive to stimuli | Nervous system disorders | 7 | 5.79 | 2.73 | 12.25 | 5.72 | 27.07 | 0.80 | 3.03 | 9.3E-05 |
| Growth retardation | Musculoskeletal and connective tissue disorders | 7 | 5.70 | 2.69 | 12.06 | 5.63 | 26.48 | 0.79 | 2.98 | 0.000126 |
| Facial paralysis | Nervous system disorders | 6 | 27.33 | 11.99 | 62.27 | 27.01 | 143.59 | 1.38 | 12.97 | 2.06E-30 |
| Autism spectrum disorder | Psychiatric disorders | 6 | 8.30 | 3.69 | 18.67 | 8.21 | 37.52 | 0.90 | 4.11 | 4.29E-07 |
| Amnesia | Nervous system disorders | 6 | 6.46 | 2.87 | 14.51 | 6.39 | 27.03 | 0.74 | 3.22 | 9.48E-05 |
| Blood pressure increased | Investigations | 6 | 4.60 | 2.05 | 10.32 | 4.55 | 16.56 | 0.48 | 2.30 | 0.022327 |
| Restlessness | Psychiatric disorders | 6 | 4.02 | 1.79 | 9.03 | 3.99 | 13.38 | 0.37 | 2.02 | 0.120574 |
| Eye movement disorder | Eye disorders | 5 | 7.28 | 3.00 | 17.67 | 7.22 | 26.50 | 0.62 | 3.40 | 0.000125 |
| Social avoidant behaviour | Psychiatric disorders | 5 | 7.09 | 2.92 | 17.20 | 7.03 | 25.57 | 0.61 | 3.31 | 0.000202 |
| Blood alkaline phosphatase increased | Investigations | 5 | 6.95 | 2.87 | 16.87 | 6.89 | 24.92 | 0.60 | 3.25 | 0.000282 |
| Body temperature increased | Investigations | 5 | 6.77 | 2.79 | 16.43 | 6.71 | 24.07 | 0.58 | 3.17 | 0.000438 |
| Obsessive-compulsive disorder | Psychiatric disorders | 5 | 6.21 | 2.56 | 15.05 | 6.16 | 21.40 | 0.53 | 2.91 | 0.001768 |
| Screaming | General disorders and administration site conditions | 5 | 5.99 | 2.47 | 14.52 | 5.94 | 20.36 | 0.50 | 2.81 | 0.003028 |
| Personality change | Psychiatric disorders | 5 | 5.67 | 2.34 | 13.75 | 5.63 | 18.87 | 0.47 | 2.66 | 0.00662 |
| Nervousness | Psychiatric disorders | 5 | 5.08 | 2.10 | 12.30 | 5.04 | 16.07 | 0.39 | 2.39 | 0.028945 |
| Visual field defect | Eye disorders | 4 | 17.46 | 6.43 | 47.40 | 17.33 | 59.77 | 0.69 | 7.31 | 5.04E-12 |
| Choking | Respiratory, thoracic and mediastinal disorders | 4 | 11.85 | 4.38 | 32.01 | 11.76 | 38.61 | 0.58 | 5.02 | 2.45E-07 |
| Bipolar disorder | Psychiatric disorders | 4 | 8.93 | 3.31 | 24.06 | 8.86 | 27.51 | 0.47 | 3.81 | 7.4E-05 |
| Respiratory rate increased | Investigations | 4 | 8.57 | 3.18 | 23.08 | 8.50 | 26.12 | 0.45 | 3.66 | 0.000151 |
| Petit mal epilepsy | Nervous system disorders | 4 | 5.90 | 2.20 | 15.87 | 5.86 | 16.00 | 0.26 | 2.54 | 0.030008 |
| Affect lability | Psychiatric disorders | 4 | 5.62 | 2.09 | 15.10 | 5.58 | 14.91 | 0.23 | 2.42 | 0.05323 |
| Impulsive behaviour | Psychiatric disorders | 4 | 5.43 | 2.02 | 14.60 | 5.40 | 14.22 | 0.21 | 2.34 | 0.077037 |
| Wolff-Parkinson-White syndrome | Cardiac disorders | 3 | 75.60 | 22.63 | 252.63 | 75.15 | 194.17 | 0.38 | 24.27 | 1.85E-41 |
| Gilbert's syndrome | Congenital, familial and genetic disorders | 3 | 66.88 | 20.18 | 221.69 | 66.47 | 173.47 | 0.39 | 21.90 | 6.1E-37 |
| Frustration tolerance decreased | Psychiatric disorders | 3 | 20.45 | 6.44 | 64.91 | 20.33 | 53.28 | 0.31 | 7.49 | 1.37E-10 |
| Cardiac murmur | Investigations | 3 | 15.95 | 5.05 | 50.39 | 15.86 | 40.66 | 0.27 | 5.90 | 8.59E-08 |
| Dysphemia | Psychiatric disorders | 3 | 11.82 | 3.76 | 37.21 | 11.76 | 28.95 | 0.20 | 4.42 | 3.51E-05 |

**Supplementary Table S10. Positive ADR signals associated with methylphenidate in oral extended-release formulations.**

| PT | SOC | Case reports | ROR | 95%CI | | PRR | χ^2^ | IC025 | EBGM05 | *P*_adj (Bonferroni) |
| --- | --- | --- | --- | --- | --- | --- | --- | --- | --- | --- |
|  |  |  |  | lower | upper |  |  |  |  |  |
| Aggression | Psychiatric disorders | 132 | 4.50 | 3.76 | 5.38 | 4.28 | 326.58 | 1.77 | 3.60 | 2.52E-70 |
| Decreased appetite | Metabolism and nutrition disorders | 120 | 5.11 | 4.24 | 6.17 | 4.88 | 361.18 | 1.93 | 4.05 | 7.36E-78 |
| Suicidal ideation | Psychiatric disorders | 96 | 3.40 | 2.76 | 4.18 | 3.29 | 151.56 | 1.36 | 2.72 | 3.73E-32 |
| Insomnia | Psychiatric disorders | 93 | 4.22 | 3.41 | 5.21 | 4.07 | 211.61 | 1.64 | 3.34 | 2.89E-45 |
| Abnormal behaviour | Psychiatric disorders | 91 | 3.37 | 2.73 | 4.17 | 3.27 | 142.02 | 1.34 | 2.69 | 4.54E-30 |
| Depression | Psychiatric disorders | 86 | 2.84 | 2.28 | 3.53 | 2.76 | 96.35 | 1.10 | 2.27 | 4.56E-20 |
| Agitation | Psychiatric disorders | 77 | 3.53 | 2.80 | 4.44 | 3.43 | 130.84 | 1.37 | 2.78 | 1.27E-27 |
| Growth retardation | Musculoskeletal and connective tissue disorders | 62 | 12.79 | 9.82 | 16.66 | 12.45 | 598.17 | 2.91 | 9.19 | 2E-129 |
| Tic | Psychiatric disorders | 61 | 6.62 | 5.10 | 8.59 | 6.46 | 269.66 | 2.14 | 4.99 | 6.36E-58 |
| Hallucination | Psychiatric disorders | 59 | 4.13 | 3.18 | 5.37 | 4.04 | 132.10 | 1.53 | 3.17 | 6.73E-28 |
| Chest pain | General disorders and administration site conditions | 57 | 3.93 | 3.01 | 5.13 | 3.85 | 117.75 | 1.46 | 3.02 | 9.31E-25 |
| Heart rate increased | Investigations | 44 | 3.81 | 2.81 | 5.15 | 3.75 | 86.68 | 1.35 | 2.85 | 6.05E-18 |
| Anger | Psychiatric disorders | 43 | 3.06 | 2.25 | 4.15 | 3.02 | 57.09 | 1.06 | 2.30 | 1.97E-11 |
| Psychotic disorder | Psychiatric disorders | 41 | 5.80 | 4.23 | 7.95 | 5.71 | 153.20 | 1.86 | 4.23 | 1.64E-32 |
| Irritability | Psychiatric disorders | 41 | 2.69 | 1.97 | 3.67 | 2.65 | 41.69 | 0.88 | 2.02 | 5.06E-08 |
| Psychomotor hyperactivity | Nervous system disorders | 39 | 3.93 | 2.85 | 5.42 | 3.87 | 81.16 | 1.36 | 2.90 | 9.82E-17 |
| Disturbance in attention | Nervous system disorders | 38 | 3.07 | 2.22 | 4.25 | 3.03 | 50.96 | 1.03 | 2.28 | 4.47E-10 |
| Feeling abnormal | General disorders and administration site conditions | 37 | 2.70 | 1.94 | 3.75 | 2.67 | 38.13 | 0.86 | 2.00 | 3.14E-07 |
| Palpitations | Cardiac disorders | 34 | 4.80 | 3.40 | 6.78 | 4.74 | 97.17 | 1.56 | 3.45 | 3E-20 |
| Hallucination, visual | Psychiatric disorders | 31 | 5.53 | 3.85 | 7.94 | 5.46 | 108.84 | 1.69 | 3.91 | 8.3E-23 |
| Restlessness | Psychiatric disorders | 28 | 4.44 | 3.04 | 6.49 | 4.40 | 71.34 | 1.40 | 3.12 | 1.42E-14 |
| Arrhythmia | Cardiac disorders | 26 | 6.37 | 4.29 | 9.47 | 6.31 | 111.06 | 1.78 | 4.36 | 2.72E-23 |
| Blood pressure increased | Investigations | 23 | 4.15 | 2.73 | 6.29 | 4.11 | 52.70 | 1.23 | 2.83 | 1.84E-10 |
| Hallucination, auditory | Psychiatric disorders | 22 | 3.78 | 2.47 | 5.79 | 3.75 | 43.31 | 1.10 | 2.57 | 2.21E-08 |
| Paranoia | Psychiatric disorders | 19 | 5.76 | 3.63 | 9.13 | 5.72 | 71.00 | 1.51 | 3.75 | 1.69E-14 |
| Delusion | Psychiatric disorders | 15 | 7.42 | 4.40 | 12.50 | 7.37 | 78.34 | 1.61 | 4.55 | 4.11E-16 |
| Withdrawal syndrome | General disorders and administration site conditions | 15 | 4.05 | 2.42 | 6.78 | 4.03 | 33.21 | 1.00 | 2.56 | 3.91E-06 |
| Raynaud's phenomenon | Vascular disorders | 12 | 20.26 | 11.02 | 37.25 | 20.15 | 189.71 | 2.09 | 10.59 | 1.74E-40 |
| Tourette's disorder | Congenital, familial and genetic disorders | 12 | 11.11 | 6.16 | 20.06 | 11.06 | 101.38 | 1.74 | 6.27 | 3.59E-21 |
| Thinking abnormal | Psychiatric disorders | 12 | 4.44 | 2.50 | 7.91 | 4.42 | 30.80 | 0.96 | 2.66 | 1.35E-05 |
| Social avoidant behaviour | Psychiatric disorders | 12 | 3.97 | 2.23 | 7.06 | 3.95 | 25.72 | 0.85 | 2.39 | 0.000187 |
| Initial insomnia | Psychiatric disorders | 11 | 5.47 | 2.99 | 10.01 | 5.45 | 38.39 | 1.10 | 3.18 | 2.74E-07 |
| Petit mal epilepsy | Nervous system disorders | 11 | 3.80 | 2.08 | 6.92 | 3.78 | 21.91 | 0.75 | 2.24 | 0.001352 |
| Eye movement disorder | Eye disorders | 11 | 3.73 | 2.04 | 6.80 | 3.71 | 21.25 | 0.73 | 2.20 | 0.001906 |
| Trichotillomania | Psychiatric disorders | 10 | 15.68 | 8.13 | 30.24 | 15.61 | 122.39 | 1.76 | 8.12 | 8.98E-26 |
| Peripheral coldness | Vascular disorders | 10 | 5.46 | 2.90 | 10.29 | 5.44 | 34.82 | 1.03 | 3.10 | 1.71E-06 |
| Self-injurious ideation | Psychiatric disorders | 10 | 4.44 | 2.36 | 8.35 | 4.42 | 25.66 | 0.84 | 2.54 | 0.000192 |
| Diplopia | Eye disorders | 10 | 3.82 | 2.03 | 7.16 | 3.80 | 20.10 | 0.69 | 2.20 | 0.003478 |
| Blindness | Eye disorders | 10 | 3.50 | 1.87 | 6.57 | 3.49 | 17.35 | 0.60 | 2.02 | 0.014713 |
| Henoch-schonlein purpura | Skin and subcutaneous tissue disorders | 9 | 9.51 | 4.83 | 18.73 | 9.48 | 63.73 | 1.36 | 5.06 | 6.76E-13 |
| Disturbance in social behaviour | Psychiatric disorders | 9 | 6.93 | 3.54 | 13.56 | 6.90 | 43.21 | 1.14 | 3.77 | 2.33E-08 |
| Euphoric mood | Psychiatric disorders | 9 | 6.55 | 3.35 | 12.81 | 6.53 | 40.16 | 1.09 | 3.58 | 1.11E-07 |
| Logorrhoea | Psychiatric disorders | 9 | 5.96 | 3.05 | 11.64 | 5.94 | 35.43 | 1.02 | 3.27 | 1.25E-06 |
| Hallucinations, mixed | Psychiatric disorders | 9 | 5.79 | 2.97 | 11.30 | 5.77 | 34.03 | 0.99 | 3.18 | 2.57E-06 |
| Homicidal ideation | Psychiatric disorders | 9 | 4.23 | 2.18 | 8.23 | 4.22 | 21.45 | 0.72 | 2.36 | 0.001717 |
| Eating disorder | Psychiatric disorders | 9 | 3.64 | 1.88 | 7.07 | 3.63 | 16.71 | 0.57 | 2.04 | 0.020599 |
| Choreoathetosis | Nervous system disorders | 8 | 15.44 | 7.42 | 32.14 | 15.38 | 96.44 | 1.49 | 7.52 | 4.36E-20 |
| Angina pectoris | Cardiac disorders | 8 | 8.95 | 4.37 | 18.34 | 8.92 | 52.74 | 1.20 | 4.62 | 1.8E-10 |
| Hyperthyroidism | Endocrine disorders | 8 | 5.79 | 2.85 | 11.76 | 5.77 | 30.24 | 0.89 | 3.08 | 1.8E-05 |
| Bipolar disorder | Psychiatric disorders | 8 | 4.16 | 2.05 | 8.42 | 4.15 | 18.54 | 0.61 | 2.25 | 0.007873 |
| Underweight | Metabolism and nutrition disorders | 7 | 16.35 | 7.45 | 35.88 | 16.29 | 89.52 | 1.35 | 7.57 | 1.44E-18 |
| Delayed puberty | Endocrine disorders | 7 | 13.70 | 6.29 | 29.86 | 13.66 | 74.48 | 1.28 | 6.50 | 2.9E-15 |
| Strabismus | Eye disorders | 7 | 8.39 | 3.91 | 18.03 | 8.37 | 42.73 | 1.03 | 4.18 | 2.97E-08 |
| Weight gain poor | Metabolism and nutrition disorders | 7 | 7.06 | 3.30 | 15.11 | 7.04 | 34.44 | 0.92 | 3.56 | 2.08E-06 |
| Precocious puberty | Endocrine disorders | 7 | 6.21 | 2.91 | 13.27 | 6.19 | 29.13 | 0.83 | 3.16 | 3.2E-05 |
| Staring | Psychiatric disorders | 7 | 6.01 | 2.81 | 12.83 | 5.99 | 27.87 | 0.80 | 3.06 | 6.13E-05 |
| Priapism | Reproductive system and breast disorders | 7 | 5.03 | 2.36 | 10.72 | 5.02 | 21.73 | 0.67 | 2.59 | 0.001484 |
| Erection increased | Reproductive system and breast disorders | 6 | 12.09 | 5.24 | 27.93 | 12.06 | 55.81 | 1.04 | 5.53 | 3.77E-11 |
| Distractibility | Psychiatric disorders | 6 | 11.40 | 4.95 | 26.28 | 11.37 | 52.30 | 1.01 | 5.25 | 2.26E-10 |
| Formication | Nervous system disorders | 6 | 8.87 | 3.88 | 20.29 | 8.85 | 39.16 | 0.89 | 4.18 | 1.85E-07 |
| Schizophrenia | Psychiatric disorders | 6 | 5.82 | 2.57 | 13.21 | 5.81 | 22.91 | 0.63 | 2.83 | 0.000804 |
| Coeliac disease | Gastrointestinal disorders | 6 | 5.32 | 2.35 | 12.04 | 5.31 | 20.18 | 0.57 | 2.60 | 0.003335 |
| Frustration tolerance decreased | Psychiatric disorders | 6 | 5.11 | 2.26 | 11.57 | 5.10 | 19.07 | 0.54 | 2.50 | 0.005951 |
| Asphyxia | Respiratory, thoracic and mediastinal disorders | 6 | 4.48 | 1.98 | 10.12 | 4.47 | 15.66 | 0.44 | 2.20 | 0.035872 |
| Trismus | Musculoskeletal and connective tissue disorders | 6 | 4.43 | 1.96 | 10.01 | 4.42 | 15.39 | 0.43 | 2.18 | 0.041343 |
| Hyperventilation | Respiratory, thoracic and mediastinal disorders | 6 | 4.07 | 1.80 | 9.18 | 4.06 | 13.45 | 0.36 | 2.01 | 0.116128 |
| Left ventricular hypertrophy | Cardiac disorders | 5 | 17.97 | 7.06 | 45.77 | 17.93 | 70.43 | 0.92 | 7.28 | 2.26E-14 |
| Madarosis | Skin and subcutaneous tissue disorders | 5 | 11.46 | 4.59 | 28.61 | 11.44 | 43.86 | 0.79 | 4.94 | 1.67E-08 |
| Herpes virus infection | Infections and infestations | 5 | 8.63 | 3.49 | 21.35 | 8.62 | 31.62 | 0.67 | 3.82 | 8.88E-06 |
| Sinus arrhythmia | Cardiac disorders | 5 | 7.47 | 3.03 | 18.41 | 7.45 | 26.46 | 0.59 | 3.34 | 0.000127 |
| Obsessive thoughts | Psychiatric disorders | 5 | 7.07 | 2.87 | 17.40 | 7.06 | 24.69 | 0.56 | 3.18 | 0.000318 |
| Bruxism | Psychiatric disorders | 5 | 5.83 | 2.38 | 14.29 | 5.82 | 19.13 | 0.45 | 2.65 | 0.005787 |
| Concussion | Injury, poisoning and procedural complications | 5 | 5.83 | 2.38 | 14.29 | 5.82 | 19.13 | 0.45 | 2.65 | 0.005787 |
| Supraventricular tachycardia | Cardiac disorders | 5 | 5.00 | 2.04 | 12.22 | 4.99 | 15.37 | 0.35 | 2.29 | 0.041739 |
| Hypokinesia | Nervous system disorders | 5 | 4.43 | 1.82 | 10.81 | 4.42 | 12.82 | 0.27 | 2.04 | 0.161828 |
| Compulsions | Psychiatric disorders | 4 | 17.15 | 6.05 | 48.63 | 17.12 | 53.78 | 0.60 | 6.39 | 1.06E-10 |
| Psychotic symptom | Psychiatric disorders | 4 | 8.05 | 2.93 | 22.12 | 8.04 | 23.26 | 0.37 | 3.28 | 0.000669 |
| Extrasystoles | Cardiac disorders | 4 | 6.56 | 2.40 | 17.93 | 6.55 | 17.94 | 0.28 | 2.71 | 0.010786 |
| Tension | Psychiatric disorders | 4 | 5.97 | 2.19 | 16.28 | 5.96 | 15.82 | 0.23 | 2.48 | 0.032974 |
| Cardiac murmur | Investigations | 4 | 4.92 | 1.81 | 13.36 | 4.91 | 12.03 | 0.12 | 2.07 | 0.247579 |
| Gastric ulcer | Gastrointestinal disorders | 4 | 4.79 | 1.76 | 13.00 | 4.78 | 11.55 | 0.10 | 2.02 | 0.3203 |
| Painful erection | Reproductive system and breast disorders | 3 | 28.47 | 8.18 | 99.15 | 28.43 | 65.39 | 0.22 | 8.31 | 2.9E-13 |
| Terminal insomnia | Psychiatric disorders | 3 | 28.47 | 8.18 | 99.15 | 28.43 | 65.39 | 0.22 | 8.31 | 2.9E-13 |
| Wolff-Parkinson-White syndrome | Cardiac disorders | 3 | 17.33 | 5.20 | 57.76 | 17.31 | 40.78 | 0.19 | 5.63 | 8.06E-08 |
| Gilbert's syndrome | Congenital, familial and genetic disorders | 3 | 15.33 | 4.64 | 50.69 | 15.31 | 35.98 | 0.17 | 5.08 | 9.44E-07 |
| Derealisation | Psychiatric disorders | 3 | 10.77 | 3.32 | 34.96 | 10.76 | 24.57 | 0.10 | 3.74 | 0.00034 |
| Maculopathy | Eye disorders | 3 | 10.77 | 3.32 | 34.96 | 10.76 | 24.57 | 0.10 | 3.74 | 0.00034 |
| Onychophagia | Psychiatric disorders | 3 | 8.86 | 2.75 | 28.52 | 8.85 | 19.57 | 0.05 | 3.14 | 0.004576 |

**Supplementary Table S11. Positive ADR signals associated with methylphenidate in transdermal extended-release formulations.**

| PT | SOC | Case reports | ROR | 95%CI | | PRR | χ^2^ | IC025 | EBGM05 | *P*_adj (Bonferroni) |
| --- | --- | --- | --- | --- | --- | --- | --- | --- | --- | --- |
|  |  |  |  | lower | upper |  |  |  |  |  |
| Application site erythema | General disorders and administration site conditions | 324 | 229.40 | 192.30 | 273.67 | 193.84 | 25524.29 | 5.79 | 69.05 | 0 |
| Application site pruritus | General disorders and administration site conditions | 166 | 177.16 | 140.67 | 223.12 | 163.11 | 12105.46 | 5.40 | 61.26 | 0 |
| Application site irritation | General disorders and administration site conditions | 102 | 140.12 | 106.19 | 184.91 | 133.30 | 6734.49 | 5.00 | 53.50 | 0 |
| Application site pain | General disorders and administration site conditions | 92 | 35.16 | 27.86 | 44.38 | 33.65 | 2335.65 | 4.07 | 22.32 | 0 |
| Disturbance in attention | Nervous system disorders | 88 | 7.63 | 6.13 | 9.50 | 7.35 | 460.44 | 2.40 | 5.84 | 1.8E-99 |
| Abnormal behaviour | Psychiatric disorders | 85 | 3.19 | 2.56 | 3.97 | 3.10 | 119.49 | 1.25 | 2.54 | 3.87E-25 |
| Disturbance in social behaviour | Psychiatric disorders | 82 | 114.87 | 85.50 | 154.33 | 110.38 | 4886.69 | 4.76 | 47.72 | 0 |
| Decreased appetite | Metabolism and nutrition disorders | 76 | 3.17 | 2.52 | 4.00 | 3.09 | 106.48 | 1.23 | 2.51 | 2.74E-22 |
| Application site rash | General disorders and administration site conditions | 61 | 126.23 | 88.99 | 179.06 | 122.56 | 3851.35 | 4.54 | 48.24 | 0 |
| Psychomotor hyperactivity | Nervous system disorders | 45 | 4.63 | 3.43 | 6.26 | 4.55 | 121.34 | 1.61 | 3.45 | 1.52E-25 |
| Application site swelling | General disorders and administration site conditions | 26 | 64.43 | 40.33 | 102.93 | 63.63 | 1088.71 | 3.43 | 29.41 | 4.4E-236 |
| Application site urticaria | General disorders and administration site conditions | 22 | 230.23 | 115.82 | 457.65 | 227.80 | 1845.41 | 3.41 | 47.97 | 0 |
| Increased appetite | Metabolism and nutrition disorders | 19 | 4.29 | 2.71 | 6.79 | 4.26 | 46.09 | 1.18 | 2.84 | 5.33E-09 |
| Application site vesicles | General disorders and administration site conditions | 16 | 65.76 | 36.14 | 119.67 | 65.27 | 682.01 | 2.82 | 26.83 | 1.2E-147 |
| Application site dryness | General disorders and administration site conditions | 14 | 90.34 | 45.88 | 177.91 | 89.74 | 737.21 | 2.68 | 30.77 | 1.1E-159 |
| Impulsive behaviour | Psychiatric disorders | 14 | 4.54 | 2.66 | 7.75 | 4.52 | 37.18 | 1.08 | 2.82 | 5.1E-07 |
| Application site erosion | General disorders and administration site conditions | 12 | 162.47 | 70.11 | 376.46 | 161.53 | 870.29 | 2.47 | 36.62 | 1.3E-188 |
| Application site burn | General disorders and administration site conditions | 11 | 28.62 | 14.91 | 54.93 | 28.48 | 240.75 | 2.12 | 13.72 | 1.27E-51 |
| Iogorrhoea | Psychiatric disorders | 11 | 7.48 | 4.07 | 13.74 | 7.44 | 58.16 | 1.37 | 4.27 | 1.14E-11 |
| Application site haemorrhage | General disorders and administration site conditions | 9 | 173.82 | 64.67 | 467.18 | 173.07 | 673.66 | 2.00 | 33.35 | 7.6E-146 |
| Application site reaction | General disorders and administration site conditions | 9 | 24.83 | 12.18 | 50.61 | 24.72 | 173.13 | 1.81 | 11.60 | 7.26E-37 |
| Application site oedema | General disorders and administration site conditions | 8 | 270.26 | 81.32 | 898.21 | 269.22 | 712.64 | 1.79 | 33.10 | 2.5E-154 |
| Application site scab | General disorders and administration site conditions | 7 | 118.18 | 42.81 | 326.20 | 117.79 | 432.32 | 1.59 | 27.06 | 2.4E-93 |
| Application site exfoliation | General disorders and administration site conditions | 7 | 42.97 | 18.34 | 100.70 | 42.83 | 216.97 | 1.56 | 16.05 | 1.96E-46 |
| Application site discolouration | General disorders and administration site conditions | 7 | 22.51 | 10.10 | 50.16 | 22.44 | 122.90 | 1.45 | 9.91 | 6.94E-26 |
| Dermatitis contact | Skin and subcutaneous tissue disorders | 7 | 8.22 | 3.83 | 17.65 | 8.19 | 41.69 | 1.01 | 4.11 | 5.05E-08 |
| Negativism | Psychiatric disorders | 6 | 6.58 | 2.90 | 14.95 | 6.57 | 27.01 | 0.72 | 3.17 | 9.58E-05 |
| Application site inflammation | General disorders and administration site conditions | 5 | 134.93 | 39.03 | 466.45 | 134.61 | 331.56 | 1.01 | 24.02 | 2.08E-71 |
| Application site warmth | General disorders and administration site conditions | 5 | 74.96 | 25.10 | 223.87 | 74.78 | 234.00 | 1.04 | 19.39 | 3.78E-50 |
| Vitiligo | Skin and subcutaneous tissue disorders | 5 | 16.06 | 6.35 | 40.64 | 16.03 | 62.96 | 0.90 | 6.64 | 1E-12 |
| Abnormal loss of weight | Metabolism and nutrition disorders | 5 | 11.24 | 4.51 | 28.02 | 11.22 | 42.96 | 0.78 | 4.86 | 2.64E-08 |
| Application site papules | General disorders and administration site conditions | 4 | 269.74 | 49.38 | 1473.53 | 269.22 | 356.31 | 0.56 | 21.84 | 8.47E-77 |
| Application site dermatitis | General disorders and administration site conditions | 4 | 67.43 | 20.29 | 224.12 | 67.31 | 174.20 | 0.67 | 16.55 | 4.24E-37 |
| Application site haematoma | General disorders and administration site conditions | 3 | 134.80 | 27.19 | 668.30 | 134.61 | 198.93 | 0.12 | 17.76 | 1.69E-42 |

**Supplementary Table S12. Venn diagram details.**

| Oral Immediate-Release | Oral Extended-Release | Transdermal Extended-Release | Count | Values |
| --- | --- | --- | --- | --- |
| TRUE | TRUE | TRUE | 3 | Disturbance in attention; Abnormal behaviour; Decreased appetite |
| TRUE | TRUE | FALSE | 19 | Insomnia; Growth retardation; Blood pressure increased; Cardiac murmur; Gilbert's syndrome; Petit mal epilepsy; Heart rate increased; Bipolar disorder; Agitation; Aggression; Restlessness; Eye movement disorder; Hallucination; Social avoidant behaviour; Frustration tolerance decreased; Feeling abnormal; Hallucination, visual; Hallucination, auditory; Wolff-Parkinson-White syndrome |
| TRUE | FALSE | TRUE | 1 | Impulsive behaviour |
| FALSE | TRUE | TRUE | 3 | Psychomotor hyperactivity; Logorrhoea; Disturbance in social behaviour |
| TRUE | FALSE | FALSE | 20 | Weight decreased; Syncope; Dyskinesia; Sudden death; Fall; Unresponsive to stimuli; Facial paralysis; Autism spectrum disorder; Amnesia; Blood alkaline phosphatase increased; Body temperature increased; Obsessive-compulsive disorder; Screaming; Personality change; Nervousness; Visual field defect; Choking; Respiratory rate increased; Affect lability; Dysphemia |
| FALSE | TRUE | FALSE | 63 | Distractibility; Trismus; Initial insomnia; Tension; Euphoric mood; Hyperthyroidism; Asphyxia; Hypokinesia; Eating disorder; Schizophrenia; Hyperventilation; Delayed puberty; Herpes virus infection; Formication; Irritability; Hallucinations, mixed; Bruxism; Withdrawal syndrome; Strabismus; Compulsions; ; Paranoia; Raynaud's phenomenon; Precocious puberty; Homicidal ideation; Thinking abnormal; Staring; Left ventricular hypertrophy; Sinus arrhythmia; Underweight; Delusion; Trichotillomania; Palpitations; Angina pectoris; Chest pain; Blindness; Concussion; Supraventricular tachycardia; Henoch-Schonlein purpura; Priapism; Suicidal ideation; Madarosis; Anger; Gastric ulcer; Diplopia; Extrasystoles; Coeliac disease; Depression; Erection increased; Tourette's disorder; Psychotic disorder; Derealisation; Arrhythmia; Psychotic symptom; Peripheral coldness; Weight gain poor; Terminal insomnia; Tic; Self-injurious ideation; Maculopathy; Painful erection; Onychophagia; Choreoathetosis; Obsessive thoughts |
| FALSE | FALSE | TRUE | 27 | Application site scab; Application site papules; Application site inflammation; Application site vesicles; Application site swelling; Vitiligo; Application site erosion; Application site discolouration; Application site haemorrhage; Application site oedema; Application site dermatitis; Application site erythema; Abnormal loss of weight; Dermatitis contact; Application site pruritus; Increased appetite; Application site urticaria; Application site haematoma; Application site irritation; Application site rash; Application site reaction; Application site exfoliation; Application site dryness; Application site pain; Application site burn; Application site warmth; Negativism |
